# Why is early-onset atrial fibrillation uncommon in patients with Duchenne muscular dystrophy? Insights from the *mdx* mouse

**DOI:** 10.1093/cvr/cvae022

**Published:** 2024-01-25

**Authors:** My-Nhan Nguyen, Charlotte Hooper, Matilde Stefanini, Besarte Vrellaku, Ricardo Carnicer, Matthew J Wood, Jillian N Simon, Barbara Casadei

**Affiliations:** Division of Cardiovascular Medicine, Radcliffe Department of Medicine, John Radcliffe Hospital, University of Oxford, L6 West Wing, Oxford OX3 9DU, UK; Division of Cardiovascular Medicine, Radcliffe Department of Medicine, John Radcliffe Hospital, University of Oxford, L6 West Wing, Oxford OX3 9DU, UK; Division of Cardiovascular Medicine, Radcliffe Department of Medicine, John Radcliffe Hospital, University of Oxford, L6 West Wing, Oxford OX3 9DU, UK; Division of Cardiovascular Medicine, Radcliffe Department of Medicine, John Radcliffe Hospital, University of Oxford, L6 West Wing, Oxford OX3 9DU, UK; Division of Cardiovascular Medicine, Radcliffe Department of Medicine, John Radcliffe Hospital, University of Oxford, L6 West Wing, Oxford OX3 9DU, UK; Department of Paediatrics and Muscular Dystrophy UK Oxford Neuromuscular Centre, University of Oxford, Oxford, UK; Division of Cardiovascular Medicine, Radcliffe Department of Medicine, John Radcliffe Hospital, University of Oxford, L6 West Wing, Oxford OX3 9DU, UK; Division of Cardiovascular Medicine, Radcliffe Department of Medicine, John Radcliffe Hospital, University of Oxford, L6 West Wing, Oxford OX3 9DU, UK

**Keywords:** Atrial fibrillation, Nitric oxide, Dystrophin, Duchenne muscular dystrophy, NOS1

## Abstract

**Aims:**

A reduction in both dystrophin and neuronal nitric oxide synthase (NOS1) secondary to microRNA-31 (miR-31) up-regulation contributes to the atrial electrical remodelling that underpins human and experimental atrial fibrillation (AF). In contrast, patients with Duchenne muscular dystrophy (DMD), who lack dystrophin and NOS1 and, at least in the skeletal muscle, have raised miR-31 expression, do not have increase susceptibility to AF in the absence of left ventricular (LV) dysfunction. Here, we investigated whether dystrophin deficiency is also associated with atrial up-regulation of miR-31, loss of NOS1 protein, and increased AF susceptibility in young *mdx* mice.

**Methods and results:**

Echocardiography showed normal cardiac structure and function in 12–13 weeks *mdx* mice, with no indication by assay of hydroxyproline that atrial fibrosis had developed. The absence of dystrophin in *mdx* mice was accompanied by an overall reduction in syntrophin and a lower NOS1 protein content in the skeletal muscle and in the left atrial and ventricular myocardium, with the latter occurring alongside reduced Nos1 transcript levels (exons 1–2 by quantitative polymerase chain reaction) and an increase in NOS1 polyubiquitination [assessed using tandem polyubiquitination pulldowns; *P* < 0.05 vs. wild type (WT)]. Neither the up-regulation of miR-31 nor the substantial reduction in NOS activity observed in the skeletal muscle was present in the atrial tissue of *mdx* mice. At difference with the skeletal muscle, the *mdx* atrial myocardium showed a reduction in the constitutive NOS inhibitor, caveolin-1, coupled with an increase in NOS3 serine^1177^ phosphorylation, in the absence of differences in the protein content of other NOS isoforms or in the relative expression NOS1 splice variants. In line with these findings, transoesophageal atrial burst pacing revealed no difference in AF susceptibility between *mdx* mice and their WT littermates.

**Conclusion:**

Dystrophin depletion is not associated with atrial miR-31 up-regulation, reduced NOS activity, or increased AF susceptibility in the *mdx* mouse. Compared with the skeletal muscle, the milder atrial biochemical phenotype may explain why patients with DMD do not exhibit a higher prevalence of atrial arrhythmias despite a reduction in NOS1 content.

**Time of primary review: 26 days**

## 1. Introduction

Duchenne muscular dystrophy (DMD) is an inherited X-linked neuromuscular disorder, with an estimated prevalence of 1 in 5000 births.^1^ DMD is the result of an out-of-frame mutation in the Xq21 chromosome locus of the *DMD* gene, which causes a deficiency of the 427 kDa rod-shaped protein, dystrophin. Although the disorder is primarily known to affect skeletal muscle, cardiac complications, including left ventricular (LV) dysfunction and dilatation, are a common late manifestation of DMD.

The neuronal isoform of nitric oxide synthase (NOS1) is closely associated with dystrophin^2^ and is nearly absent in the skeletal muscle of DMD patients.^3^ Deficiency in NOS1-derived nitric oxide (NO) exacerbates the DMD phenotype by impairing skeletal muscle perfusion and contractile function.^4–6^

We have previously reported that microRNA-31 (miR-31) up-regulation in the atrial myocardium of patients and animal models with atrial fibrillation (AF) leads to translational repression of dystrophin and accelerated decay of NOS1’s mRNA and that the resulting reduction in NOS1-derived NO contributes to the AF-induced atrial electrical remodelling that promotes the maintenance of the arrhythmia.^7^ In agreement with these findings, NOS1^−/−^ mice show atrial hallmark features of AF-induced electrical remodelling as well as an increased propensity to develop AF in response to atrial burst pacing.^7^

In contrast, the prevalence of AF in DMD patients is relatively low, even after they develop overt cardiomyopathy,^8^ suggesting that absence of dystrophin may not result in miR-31 up-regulation or a significant reduction in NO availability in the atrial myocardium. To test this hypothesis, we investigated miR-31 expression, NOS1 mRNA and protein content, and NO synthesis in the atrial and ventricular myocardium of *mdx* mice and examined whether dystrophin deficiency leads to an increased propensity to develop atrial arrhythmias in this model.

## 2. Methods

### 2.1 Animals

Male C57BL/10ScSn-Dmd^mdx^/J (*mdx*) mice were crossed with female C57/Bl10Snj mice to generate F1 heterozygous female and wild-type (WT) male mice. F1 heterozygous females were then crossed with WT males to generate WT, *mdx*, and heterozygous littermate mice. Male *mdx* mice and their WT littermates aged 12–13 weeks were used in all experiments. Genotyping was confirmed by following a protocol outlined by Shin *et al*.^9^ (see Supplementary material online, *Figure S1*). All animal studies were approved by the local regulatory authority and conducted in accordance with the guidelines from the UK Home Office Guidance on the Operation of Animals (Scientific Procedures) Act 1986 and Directive 2010/63/EU.

### 2.2 Quantitative real-time polymerase chain reaction

Total mRNA was isolated from murine atrial and ventricular tissues using the mirVana miRNA isolation kit (Applied Biosystems), according to manufacturer’s instructions. Template RNA (0.5 µg) was used to reverse-transcribe into cDNA using the QuantiTect Reverse Transcription Kit (Qiagen). TaqMan PreAmp Master Mix (Applied Biosystems) was used on 10 ng/µL cDNA from atrial and ventricular samples to amplify cDNA content in these sample sets using a TaqMan Assay pool of the following probes as per the manufacturer’s instructions. Quantitative polymerase chain reaction (qPCR) was performed in duplicate with QuantStudio Flex 7, TaqMan Master Mix (both Applied Biosystems), and TaqMan primers (ThermoFisher Scientific) for the following target mouse mRNA transcripts: hypoxanthine phosphoribosyltransferase 1 (HPRT1; Mm01545399_m1), peptidylprolyl isomerase A (PPIA; Mm02342430_g1), glyceraldehyde 3-phosphate dehydrogenase (GAPDH; Mm99999915_g1), myosin heavy chain 6 (Myh6; Mm00440359_m1), tropomyosin 1 (Tpm1; Mm00445895_g1), NOS1 exons 26–27 (targets all NOS1 splice variants; Mm01208059_m1), NOS1 exons 1–2 (targets NOS1-α, -µ, and -2; Mm00435171_m1), NOS1 exons 3–4 (targets all NOS1 excluding NOS1-γ; Mm00435173_m1), NOS1 exons 8–9 (targets all NOS1 excluding NOS1-2; Mm00435177_m1), NOS1 exons 16–17 (targets all NOS1 excluding NOS1-µ; Mm01208055_m1), and *Dmd* (i.e. gene that encodes dystrophin protein) at the rod domain (Mm00464475_m1), 3ʹ untranslated region (UTR; Mm00464533_m1), and 5ʹ UTR (Mm01216931_m1). Total mRNA expression was quantified using the comparative cycle threshold (2^−ΔΔCT^) method, normalized to a housekeeping gene (HPRT or PPIA) or a cardiomyocyte marker (Myh6 or Tpm1), and a reference control group. The proportion of NOS1 splice variant mRNA expression between WT and *mdx* mice was calculated from the ΔCT values of each NOS1 targeted exon probe relative to total NOS1 (exons 26–27), each of which was normalized to the most stable housekeeping gene for the respective tissue type (HPRT or GAPDH).

### 2.3 Immunoblotting

The protein content of dystrophin and NOS isoforms in murine atrial and ventricular homogenates was evaluated by immunoblotting (IB). Protein was extracted in homogenizing buffer (RIPA buffer; Cell Signalling Technology), cOmplete protease, and PhosSTOP phosphatase inhibitors (Roche), separated on a 3–8% Tris-Acetate NuPAGE gel (Invitrogen) or 4–15% Mini-PROTEAN TGX stain-free gels (Bio-Rad) and transferred onto a 0.2 µm nitrocellulose membrane (Bio-Rad). The membrane was blocked in 5% skim milk/PBST and incubated overnight (at 4°C) with the following primary antibodies: antimouse N-terminus NOS1 (Santa Cruz Biotechnology, A11 Clone; 1:1000), antimouse dystrophin rod domain (NCL-DYS1; 1:2000), antimouse dystrophin C-terminal domain (NCL-DYS2, Leica Biosystems; 1:2000), antimouse syntrophin (Abcam; 1:2000), antimouse NOS3 (BD Transduction Laboratories; 1:3000), antimouse phosphorylated-NOS3-serine^1177^ (p-NOS3-ser^1177^, BD Transduction Laboratories; 1:1000), antimouse p-NOS3-threonine^495^ (p-NOS3-thr^495^, BD Transduction Laboratories; 1:1000), antimouse NOS2 (Abcam; 1:1000), antimouse caveolin-1 (BD Transduction Laboratories; 1:1000), antirabbit caveolin-1 (Abcam; 1:1000), antimouse caveolin-3 (Cav-3) (Santa Cruz; 1:1000), antirabbit connexin-43 (Cx-43, Sigma; 1:1000), antimouse heat shock protein 90 (Hsp90, Enzo; 1:500), antimouse NOS1AP (Santa Cruz; 1:1000), antirabbit sarcomeric alpha-actinin (Abcam; 1:5000), and GAPDH (Millipore; 1:50000) or horseradish peroxidase (HRP)-conjugated GAPDH (GAPDH-HRP, Promega; 1:25 000). With the exception of GAPDH-HRP, the membrane was incubated with the relevant HRP-conjugated secondary antibodies (all from Promega). Protein bands were detected by enhanced chemiluminescence and visualized on ChemiDoc and Image Lab (v5.1, Bio-Rad).

### 2.4 Polyubiquitinated protein pulldown using tandem ubiquitin-binding entities

Atrial or skeletal muscle tissues were homogenized in CelLytic buffer (Sigma), 100 mM N-ethylmalemide (Sigma), 50 µM PR-619 (pan-deubiquitinase inhibitor; Sigma), and cOmplete protease inhibitors and centrifuged at 13 000 × g (4°C) for 10 min to collect the homogenate. High-affinity pulldowns of polyubiquitin conjugates were performed using Agarose-tandem ubiquitin-binding entities (TUBEs) (LifeSensors) according to the manufacturer’s protocol. Due to the low NOS1 protein content in *mdx* mice, a 2.5-5 fold increase in total protein input was used to normalize the NOS1 pulldown amount between genotypes. Polyubiquitinated proteins were eluted in 2× LDS and 10× reducing agent sample, separated on 3–8% Tris-Acetate NuPAGE gels and immunoblotted for antimouse NOS1. Pooled left (two per sample) and right (three per sample) atrial samples were homogenized to obtain high atrial concentrations sufficient for polyubiquitinated protein pulldowns.

### 2.5 Immunofluorescence staining

The localization of NOS1 and dystrophin was examined by immunofluorescence staining in isolated adult cardiomyocytes from both WT and *mdx* mice (isolation protocol as described by Zhang *et al*.^10^). Cells were fixed in 4% paraformaldehyde for 10 min and permeabilized in 0.2% Triton for 5 min, followed by blocking in 5% donkey serum in 0.1% Triton/PBS. Cells were incubated overnight (4°C) with the following primary antibodies: antimouse dystrophin rod domain (NCL-DYS1; 1:100), antirabbit NOS1 (ThermoFisher Scientific; 1:100), or antimouse Cav-3 (Santa Cruz; 1:300) and then the relevant Alexa Fluor secondary antibodies at room temperature. Cells were incubated with NucBlue (nuclei stain; ThermoFisher Scientific) and mounted with ProLong Diamond Antifade (Invitrogen) and visualized under a microscope (Leica) at ×63 magnification.

### 2.6 NOS activity measurement

Atrial and ventricular NOS activity was examined by measuring the conversion of [^14^C]L-arginine (Amersham) to citrulline, in the presence of an arginase inhibitor (N^ω^-hydroxy-nor-Arginine; Calbiochem), by high-performance liquid chromatography (HPLC), as described previously.^11^ Four left (LA) or right atria (RA) of the same genotype were pooled together to obtain 300 µg of protein, which was used for each measurement. Likewise, two right ventricles (RV) of the same genotype were pooled together to achieve 600 µg of RV protein. Six hundred µg of protein from one LV was used to measure NOS activity. NOS activity is reported as the N omega-nitro-L-arginine methyl ester hydrochloride (L-NAME)-inhibitable fraction of arginine-to-citrulline conversion.

### 2.7 Echocardiography

Echocardiography was performed in anaesthetized mice (4% and 2% isoflurane in O_2_ at induction and maintenance, respectively) using the Vevo 2100 Imaging Platform and a MS550D transducer (VisualSonics). B-mode loops of the short- and long-axis parasternal views of the LV were obtained. A four-chamber view of the heart was obtained to assess LV diastolic function by pulsed wave Doppler.

### 2.8 Transoesophageal atrial burst pacing

AF or atrial flutter induction was assessed by transoesophageal atrial burst pacing, as previously described.^7^ Briefly, mice were anaesthetized with isoflurane (4% and 2% in O_2_ at induction and maintenance, respectively) and placed over a temperature-controlled pad. Intradermal electrodes were inserted, and surface electrocardiogram (ECG) was recorded using a bio-amplifier (iso-DAM8A; World Precision Instruments) and Power1401 data acquisition system (Cambridge Electronic Design). To induce atrial tachyarrhythmias, a 1.7-French octapolar electrode catheter (NuMed) was inserted into the oesophagus to deliver electrical stimuli to the atria via a DS3 isolated current stimulator (Digitimer). Three sequences of 26 pacing bursts were applied, commencing at a cycle length of 60 ms, with successive 2 ms decreases in cycle length until a cycle length of 10 ms was reached. Each pacing burst delivers 40 electrical stimuli at twice the threshold for atrial capture. Once induced, we waited for spontaneous termination of an arrhythmic episode for a maximum of 20 min before reinitiating the next pacing sequence. Above 20 min, overdrive pacing was used to reestablish sinus rhythm (SR) before reinitiation of burst pacing. All mice were successfully reestablished into SR during the study. All ECG signals were recorded and analysed on Spike2 (v7.09, Cambridge Electronic Design). For quantification of pacing-induced AF, we defined an episode of AF/atrial flutter as lasting more than 2 s.

### 2.9 Collagen assay

Collagen content in WT and *mdx* atria was measured using the QuickZyme Sensitive Tissue Collagen Assay (QuickZyme Biosciences) according to the manufacturer’s protocol.

### 2.10 Statistical analysis

All data were tested for normality using the Shapiro–Wilks test. Normally distributed data were compared using the one-way ANOVA or two-way ANOVA with Bonferroni’s correction for multiple comparisons, Student’s unpaired *t*-test, or Fisher’s exact test, as appropriate, and displayed as mean ± standard deviation (SD). Nonnormally distributed data were compared using the Kruskal–Wallis test or the Mann–Whitney *U* test, as appropriate, and displayed as median ± interquartile range (IQR). A *P* value < 0.05 was considered statistically significant.

## 3. Results

### 3.1 Absence of dystrophin is associated with a moderate reduction in myocardial NOS1 without changes in miR-31 expression

The *mdx* mouse carries a nonsense point mutation in exon 23 of the *Dmd* gene that aborts full-length dystrophin expression. Accordingly, atrial (see Supplementary material online, *Figure S2B*), ventricular (see Supplementary material online, *Figure S2C*), and skeletal muscle (see Supplementary material online, *Figure S2A*) *Dmd* mRNA levels encoding for this region were significantly reduced in *mdx* mice. Likewise, expression of 5ʹ UTR and 3ʹ UTR *Dmd* mRNA, which encode the N- and C-terminal domains of dystrophin, was markedly reduced in *mdx* atria (see Supplementary material online, *Figure S2E* and *H*), ventricles (see Supplementary material online, *Figure S2F* and *I*), and skeletal muscle (see Supplementary material online, *Figure S2D* and *G*). Western blotting using antibodies directed against the dystrophin rod domain and C-terminus showed complete absence of dystrophin protein in all *mdx* muscle tissue types examined (see Supplementary material online, *Figure S2J*–*R*).

**Figure 1:**
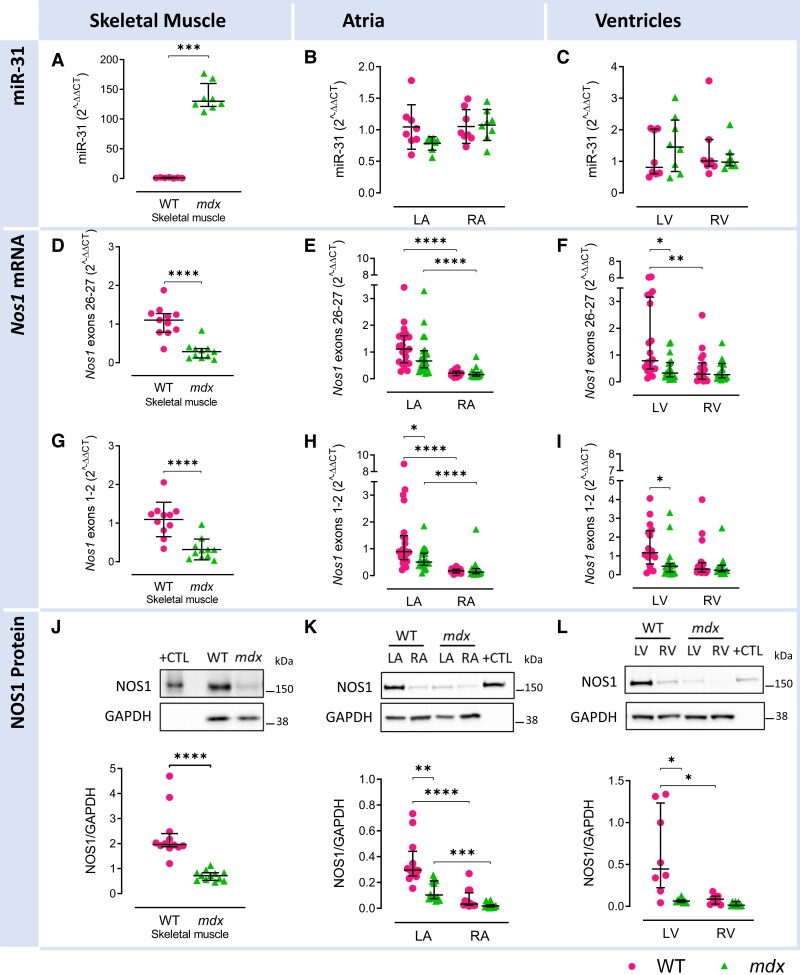
Differences in skeletal muscle, atrial, and ventricular miR-31, neuronal NOS1 transcript, and protein expression between *mdx* and WT mice. miR-31 expression in WT and *mdx* mice in (*A*) skeletal muscle, (*B*) LA and RA, and (*C*) LV and RV, *N* = 7–8 per group. mRNA expression of total *Nos1* in WT and *mdx* mice in (*D*) skeletal muscle, (*E*) LA and RA, and (*F*) LV and RV: *N* = 11 (*D*), *N* = 18–22 (*E*), and *N* = 18–20 (*F*) per group. mRNA expression of PDZ domain-containing splice variants of *Nos1* (*Nos1*-α, µ, and 2) in (*G*) skeletal muscle, (*H*) LA and RA, and (*I*) LV and RV in WT and *mdx* mice: *N* = 11 (*G*), *N* = 19–22 (*H*), and *N* = 15–18 (*I*) per group. (*J*) Densitometry analysis and representative immunoblots showing protein content of NOS1 in the skeletal muscle of WT and *mdx* mice. *N* = 12 per group. Protein homogenate (2 µg) from WT brain was used as positive control (+CTL). (*K*) Densitometry analysis and representative immunoblots showing myocardial protein content of NOS1 in the LA and RA of WT and *mdx* mice. *N* = 8 per group. Diluted (1:40) protein homogenate from WT brain was used as positive control (+CTL). (*L*) Densitometry analysis and representative immunoblots showing protein content of NOS1 in the LV and RV of WT and *mdx* mice. *N* = 8 per group. Diluted (1:50) protein homogenate from WT brain was used as positive control (+CTL). Data are expressed as mean ± SD (*B* and *G*) or median ± IQR (*A*, *C*–*F*, and *H*–*L*). *P* values were determined by Student’s unpaired *t*-test (*G*), Mann–Whitney *U* test (*A*, *D*, and *J*), two-way ANOVA with Bonferroni’s multiple comparison (*B*) on log-transformed data (*E*–*F* and *H*–*I*), or Kruskal–Wallis ANOVA test with Dunn’s multiple comparison (*C* and *K*–*L*). *P < 0.05, **P < 0.01, ***P < 0.001, ****P < 0.0001.

miR-31 is up-regulated in the skeletal muscle of *mdx* mice, and miR-31 inhibition enhances dystrophin mRNA translation in human DMD myoblasts when dystrophin synthesis is rescued through exon skipping.^12^ In patients with AF, atrial-specific up-regulation of miR-31 also leads to dystrophin mRNA translational repression and, independently, it accelerates the *NOS1* mRNA decay.^7^ We examined whether miR-31 expression was also increased in the myocardium of *mdx* mice and if NOS1 expression was altered by consequence. In keeping with previous reports,^12^ we observed a >100-fold up-regulation of miR-31 in *mdx* skeletal muscle, relative to WT (*Figure 1A*). In contrast, we found no evidence of atrial or ventricular miR-31 up-regulation in dystrophin-deficient hearts (*Figure 1B* and *C*). There was also no difference in miR-31 expression between left and right cardiac chambers in either genotype (*Figure 1B* and *C*).

*Nos1* mRNA levels were examined using two different TaqMan probes: one that targets *Nos1* exons 26–27 and amplified all *Nos1* splice variants (total *Nos1*) and one that targeted exons 1–2 and amplified only *Nos1* splice variants that contained the PDZ domain important for anchoring NOS1 protein to the dystrophin complex (*Nos1*-α, -µ, and -2).^3^ In both WT and *mdx* mice, gene expression of total and PDZ domain-containing *Nos1* isoforms was significantly lower in the RA compared with the LA (*Figure 1E* and *H*). Total atrial *Nos1* transcript levels did not differ significantly between genotypes (*Figure 1E*), whereas *Nos1* splice variants (*Nos1*-α, -µ, and -2) encoding the PDZ domain were significantly reduced in the LA of *mdx* mice compared with WT (*Figure 1H*). These chamber-specific differences and the trends between genotypes were conserved when *Nos1* transcripts were normalized to cardiomyocyte-specific markers (Tpm1 and Myh6; see Supplementary material online, *Figure S3A*–*D*), the expression of which did not differ between genotypes or chambers.

Chamber differences in *Nos1* expression were also found in the ventricles of WT mice, with *Nos1* content greater in the LV compared with RV, whereas *mdx* mice showed a reduction in LV total and PDZ domain-containing *Nos1* isoforms sufficient to abolish the difference between heart chambers (*Figure 1F* and *I*). Similar to the LV myocardium, the skeletal muscle of *mdx* mice showed a significant reduction in total and PDZ domain *Nos1* mRNA (*Figure 1D* and *G*).

NOS1 protein content mirrored the expression patterns found at the transcript level, with *mdx* mice showing reduced LA, LV, and skeletal muscle NOS1 content compared with WT mice, as well as higher NOS1 protein level in the WT left chambers (atrium and ventricle) compared with the right where NOS1 did not differ between genotypes (*Figure 1J–L*).

**Figure 2:**
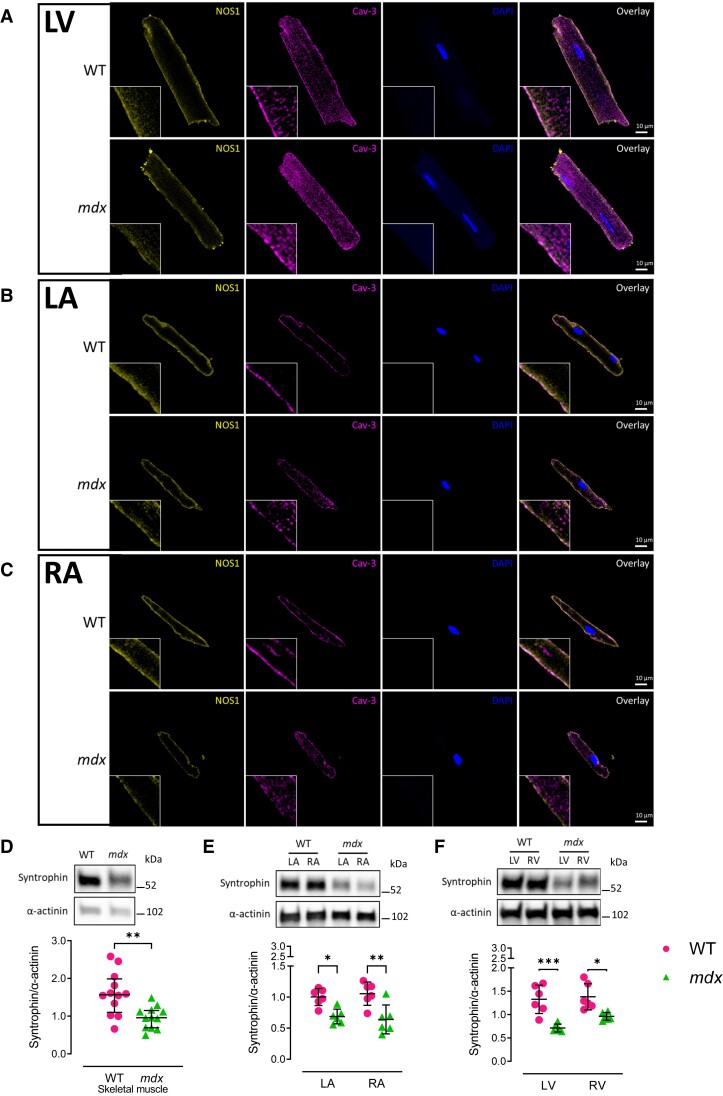
NOS1 localization and the abundance of syntrophin in the *mdx* mice. (*A*–*C*) Representative immunofluorescence staining of NOS1 (yellow), Cav-3 (magenta), and DAPI (blue) in isolated adult cardiomyocytes from WT and *mdx* LV, LA, and RA. NOS1 localizes to the sarcolemma membrane in WT and *mdx* mice; however, this staining is reduced in the *mdx* myocardium. Cav-3 was used as a sarcolemma membrane marker. (*D*–*F*) Representative immunoblots and densitometry analysis of syntrophin and α-actinin in WT and *mdx* skeletal muscle, LA, RA, LV, and RV. α-actinin was used as housekeeping control for all immunoblots. *N* = 12 (*D*) and *N* = 6 (*E* and *F*) per group. Data are expressed as mean ± SD. *P* values were determined by Student’s unpaired *t*-test (*D*) or two-way ANOVA with Bonferroni’s multiple comparison (*E* and *F*). *P < 0.05, **P < 0.01, ***P < 0.001.

As expected, immunofluorescence staining confirmed loss of dystrophin protein in both atrial and LV adult cardiomyocytes of *mdx* mice (see Supplementary material online, *Figure S4A*–*C*). NOS1 protein was detected at the sarcolemma membrane in the atria and LV from WT mice (*Figure 2A–C*), and while its presence was still observed in *mdx* cardiomyocytes, its expression was markedly reduced (*Figure 2A–C*).

**Figure 3:**
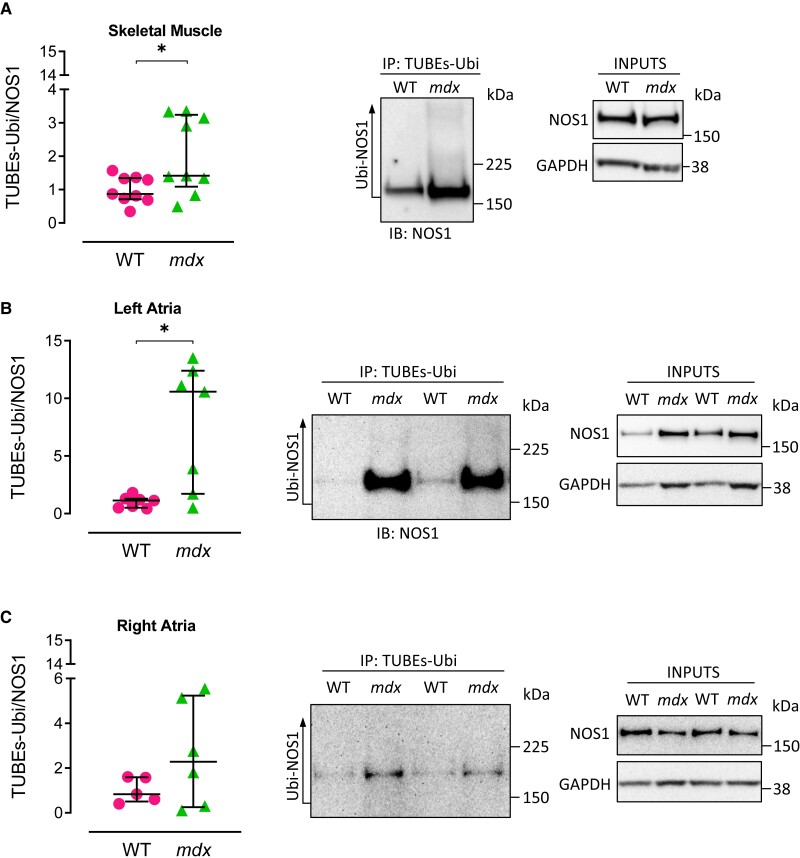
Ubiquitination of NOS1 in the absence of dystrophin. Ubiquitinated NOS1 was pulled down by immunoprecipitation (IP) with TUBEs-Ubi, followed by IB with NOS1, in WT and *mdx* skeletal muscle (*A*, *N* = 9/group), LA (*B*, *N* = 7 pooled samples/group), and RA (*C*, *N* = 5–6 pooled samples/group). Ubiquitinated NOS1 was normalized to input (10% of lysate). For atrial experiments, two LA were pooled together for each sample. Similarly, three RA were pooled together for each sample. Data are expressed as median ± IQR. *P* values were determined by Mann–Whitney *U* test. *P < 0.05.

In cardiac tissue, NOS1 anchoring to the dystrophin–glycoprotein complex has been shown to depend on α-syntrophin binding.^13^ As such, we examined whether myocardial syntrophin protein levels were reduced in *mdx* mice and found this to be the case in both the atria (*Figure 2E*) and the LV (*Figure 2F*), although in contrast with NOS1, there was no evidence for chamber-specific differences. In line with previous reports,^14^ we also found a reduction in skeletal muscle syntrophin levels in *mdx* mice as compared with their WT littermates (*Figure 2D*). In addition, NOS1 was found to be highly polyubiquitinated in the *mdx* skeletal muscle and LA (*Figure 3*), suggesting that, along with the reduction in *Nos1* mRNA, increased proteasomal degradation of membrane-displaced NOS1 may account for the lower NOS1 protein content observed.

Overall, these findings indicate that while dystrophin depletion in the skeletal muscle of *mdx* mice is associated with up-regulation of miR-31 and decreased *Nos1* expression, this association is not maintained in myocardial tissue. Instead, atria and ventricles from *mdx* hearts show unperturbed miR-31 expression, despite clear evidence for disassembly of the dystrophin–glycoprotein complex and reduced *Nos1* mRNA level and protein content in the LA and LV chambers.

### 3.2 Dystrophin and NOS1 protein depletion does not increase AF vulnerability

We then investigated whether dystrophin deficiency and the resultant depletion of NOS1 at the sarcolemma was sufficient to increase atrial arrhythmia susceptibility in *mdx* mice, despite the absence of miR-31 up-regulation.

We first confirmed by echocardiography that the young adult *mdx* mice (which we used in all protocols) showed no evidence of the cardiomyopathy they develop later in life.^15^  *mdx* mice aged 12–13 weeks showed no evidence of cardiac remodelling or LV systolic dysfunction on echocardiography (*Table 1* and see Supplementary material online, *Figure S5*). LV diastolic function was also preserved in *mdx* mice (*Table 1*). Atrial collagen content was significantly higher in the right chambers compared with the left but not significantly different between genotypes (see Supplementary material online, *Figure S6*). There were no differences noted across ECG parameters between genotypes (see Supplementary material online, *Table S1*) and no incidence of sudden death in *mdx* mice during the study period.

**Table 1:** Echocardiographic parameters in wild-type and *mdx* mice

	WT	*mdx*	P value
*N*	10	12	
Age (weeks)	12.41 ± 0.31	12.20 ± 0.21	0.08
Systolic function			
Heart rate (b.p.m.)	480.90 ± 23.62	480.00 ± 40.77	0.95
LVESV (µL)	13.33 ± 3.55	13.24 ± 6.19	0.97
LVEDV (µL)	51.31 ± 8.37	49.74 ± 9.28	0.68
LVIDs (mm)	2.02 ± 0.21	1.99 ± 0.37	0.77
LVIDd (mm)	3.50 ± 0.24	3.45 ± 0.27	0.65
LVPWs (mm)	1.44 ± 0.24	1.50 ± 0.18	0.45
LVPWd (mm)	0.94 ± 0.15	0.94 ± 0.14	0.88
EF (%)	79.78 ± 6.64	74.21 ± 9.34	0.90
FS (%)	42.16 ± 5.83	42.82 ± 7.69	0.83
Diastolic function			
Heart rate (b.p.m.)	341.00 ± 22.83	360.40 ± 41.91	0.21
E/A ratio	1.60 ± 0.19	1.58 ± 0.24	0.82
E/E′ ratio	32.99 ± 3.52	34.55 ± 8.03	0.57
IVRT (ms)	16.15 ± 4.980	17.24 ± 4.993	0.61
Deceleration time (ms)	28.25 ± 5.94	29.68 ± 4.18	0.50

Data presented as mean ± SD. *P* values determined by Student’s unpaired *t*-test.

µL, microlitre; EF, ejection fraction; FS, fractional shortening; IVRT, isovolumic relaxation time; LVEDV, left ventricular end-diastolic volume; LVESV, left ventricular end-systolic volume; LVIDd, left ventricular internal dimension at diastole; LVIDs, left ventricular internal dimension at systole; LVPWd, left ventricular posterior wall at diastole; LVPWs, left ventricular posterior wall at systole; ms, milliseconds.

**Figure 4:**
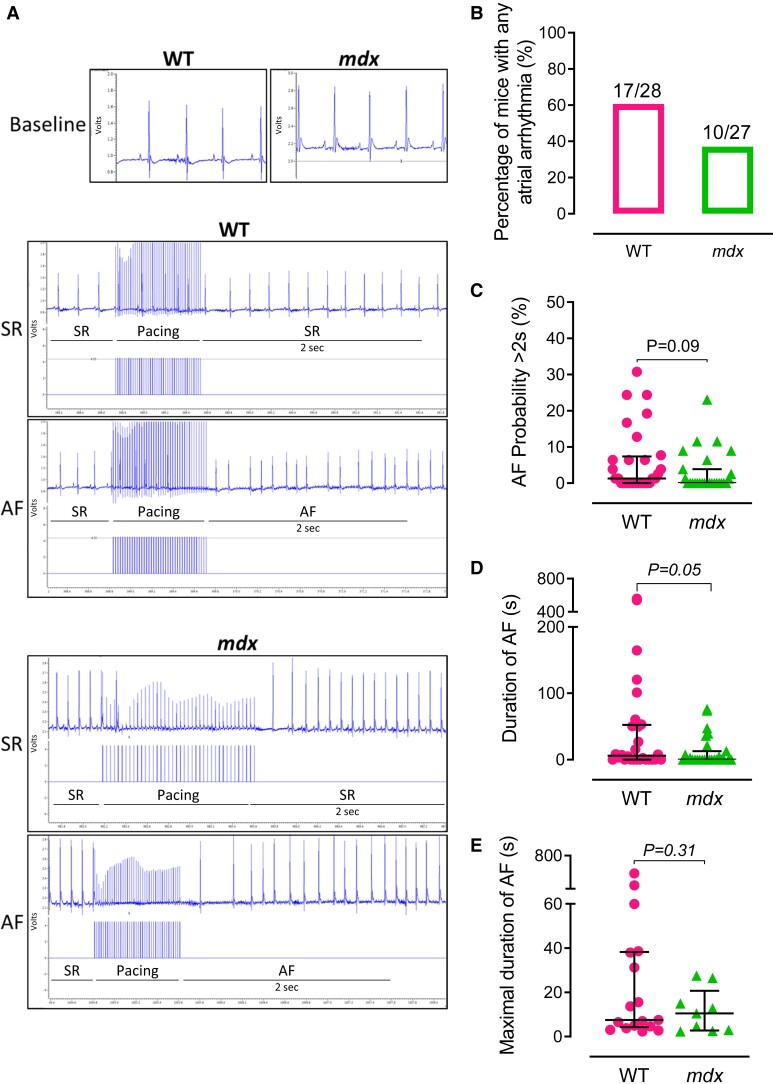
AF induction was not increased in *mdx* mice. (*A*) Representative ECG traces in WT and *mdx* mice at baseline and ECGs showing SR and AF lasting more than 2 s after atrial burst pacing. (*B*) Proportion of WT (*N* = 28) and *mdx* (*N* = 27) mice with any atrial arrhythmia induced by atrial burst pacing *in vivo*. (*C* and *D*) Probability and average duration of AF induction in WT and *mdx* mice. (*E*) Maximal duration of AF induction in a subset of WT (*N* = 17) and *mdx* (*N* = 9) mice exhibiting atrial arrhythmias. Data are expressed as percentages (*B*) or as median ± IQR (*C–E*). *P* values were determined by Fisher’s exact test (*B*) or Mann–Whitney *U* test (*C–E*).

Transoesophageal burst pacing was used to assess the susceptibility to atrial arrhythmias in WT and *mdx* mice (*Figure 4A*). As shown in *Figure 4B*, there were no significant differences in AF/atrial flutter inducibility between genotypes, although there was a trend for *mdx* mice to have a lower rate of pacing-induced atrial arrhythmias. Similarly, *mdx* mice were less likely to have atrial arrhythmias lasting more than 2 s (*P* = 0.09; *Figure 4C*) and more likely to have a shorter atrial arrhythmias duration overall (*P = 0.05*; *Figure 4D*). There were no differences in the maximal duration of AF between genotypes (*Figure 4E*).

Together, these results show that absence of dystrophin and the accompanying reduction in NOS1 protein content in the LA, in the absence of changes in cardiac miR-31 expression, are not sufficient to increase arrhythmia susceptibility in *mdx* mice.

### 3.3 Despite the depletion of LA NOS1 in dystrophin-deficient hearts, NOS activity remains unchanged

AF is associated with a significant reduction in both NOS1 protein and total NOS activity in both atria.^7^ In contrast, a previous report has suggested that NOS1 depletion in the myocardium of young *mdx* mice may be compensated for by an increase in the activity or expression of other NOS isoforms resulting in preserved NO synthesis.^16^

**Figure 5:**
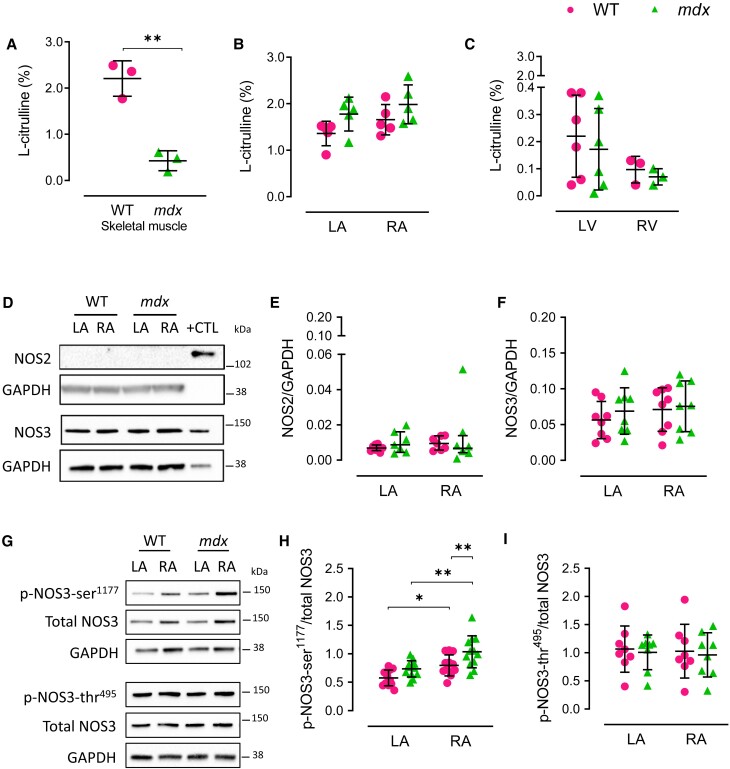
NOS activity and the atrial protein content and phosphorylation status of other NOS isoforms. (*A*) Skeletal muscle NOS activity in WT and *mdx* mice. *N* = 3 per group. (*B*) Atrial NOS activity in WT and *mdx* mice. *N* = 5 per group. NOS activity was measured in pooled samples, each containing RA or LA from 4 mice of the same genotype. (*C*) LV and RV NOS activity in WT and *mdx* mice. *N* = 3–6 per group. NOS activity in the RV was measured from pooled samples containing two mice of the same genotype. (*D*–*F*). Representative immunoblots and densitometry analysis showing LA and RA protein content of inducible NOS (NOS2) and endothelial NOS (NOS3) in WT and *mdx* mice. *N* = 7–8 per group. Lipopolysaccharide-activated macrophages and WT lung tissue were used as positive controls (+CTL) for NOS2 and NOS3 protein content, respectively. (*G*–*I*) Representative immunoblots and densitometry analysis of phosphorylated NOS3 in atrial protein homogenates of WT and *mdx* mice. Phosphorylation of NOS3 was examined at serine^1177^ (*P*-NOS3-ser^1177^) and threonine^495^ (p-NOS3-thr^495^); *N* = 8–12 per group. Data are expressed as mean ± SD (*A*–*C*, *F*, and *H*–*I*) or median ± IQR (*E*). *P* values were determined by Student’s unpaired *t*-test (A), two-way ANOVA with Bonferroni’s multiple comparison (*B*–*C*, *F*, and *H*–*I*) or Kruskal–Wallis ANOVA test with Dunn’s multiple comparison. (*E*) *P < 0.05, **P < 0.01.

Relative to WT, the *mdx* skeletal muscle showed an 81% reduction (*P < 0.002*) in NO synthesis, as measured by the L-NAME-inhibitable fraction of the L-arginine-to-citrulline conversion (*Figure 5A*). In contrast, despite the difference in NOS1 protein level between sides and genotypes, atrial and ventricular NOS activity did not differ significantly between WT and *mdx* mice nor did we find differences in NOS activity between LA and RA or LV and RV chambers in either genotype (*Figure 5B* and *C*).

We then investigated whether compensatory up-regulation of other NOS isoforms or relative changes in *Nos1* splice variant expression accounted for the preservation of myocardial NOS activity in *mdx* mice. Protein content of the inducible NOS isoform (NOS2) was negligible in atrial and ventricular chambers of WT and *mdx* mice (*Figure 5D* and *E* and see Supplementary material online, *Figure S7A* and *B*). There were also no chamber- or genotype-dependent differences in myocardial protein content of the endothelial NOS isoform (NOS3; *Figure 5F* and see Supplementary material online, *Figure S7C*). Since phosphorylation of NOS3 is known to regulate its enzymatic activity,^17^ myocardial NOS3 serine 1177 phosphorylation (p-NOS3-ser^1177^) was compared between genotypes and found to be significantly higher in the RA than in the LA of both genotypes and in the RA of *mdx* mice compared with WT littermates (*Figure 5G* and *H*). However, this was not the case in the *mdx* LA or LV (see Supplementary material online, *Figure*  S7*D* and *E*). In contrast, NOS3 phosphorylation at threonine 495 (p-NOS3-thr^495^) was not different in *mdx* atria or ventricles relative to side or genotype (*Figure 5I* and see Supplementary material online, *Figure S7F*).

**Figure 6:**
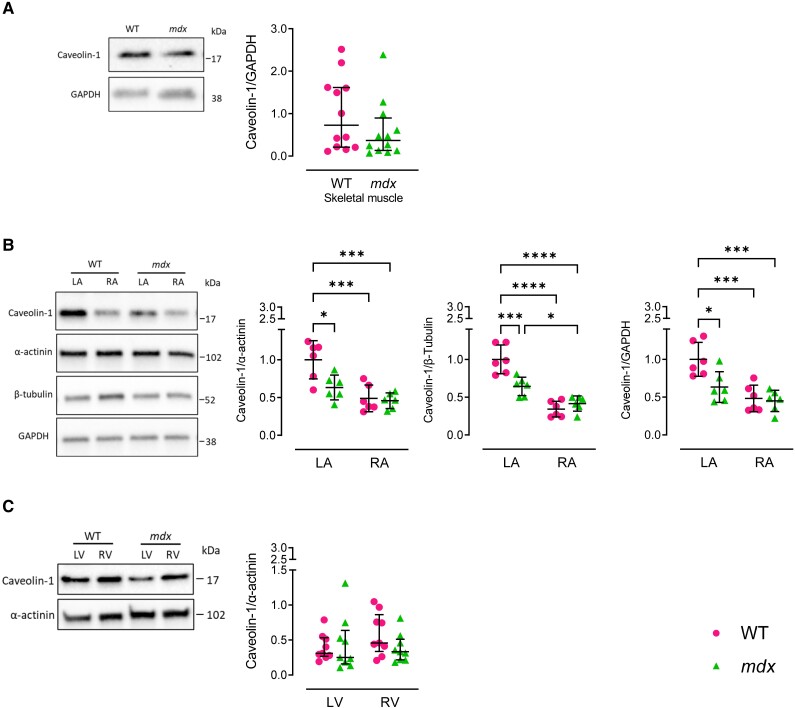
LA protein expression of caveolin-1 was reduced in *mdx* mice. (*A*–*C*) Representative immunoblots and densitometry analysis of caveolin-1 in skeletal muscle (*A*), LA and RA (*B*), and LV and RV (*C*) of WT and *mdx* mice. The following housekeeping controls were used, GAPDH for skeletal muscle; α-actinin, β-tubulin, and GAPDH for the atria; and α-actinin for the ventricles. *N* = 12 (*A*), *N* = 6 (*B*), and *N* = 9 (*C*) per group. Data are expressed as mean ± SD (*B*) or median ± IQR (A and *C*). *P* values were determined by Mann–Whitney *U* test (A), one-way ANOVA with Bonferroni’s multiple comparison (*B*), or Kruskal–Wallis ANOVA test with Dunn’s multiple comparison (*C*). *P < 0.05, ***P < 0.001, ****P < 0.0001.

*Nos1* can be alternatively spliced at exons within its open reading frame, giving rise to five distinct splice variants—three N-terminal PDZ domain-containing variants (*Nos1*-α, -µ, and -2) and a *Nos1*-β and -γ variant—which differ in their intracellular localization, enzymatic activity, and expression levels.^18^ Since *Nos1* splice variant qPCR probes are not commercially available, we examined the mRNA expression of the known *Nos1* splice variants using TaqMan probes, which target unique exons within the *Nos1* sequence that are absent in each of the *Nos1* splice variants (see Supplementary material online, *Figure S8A*), and used this method to calculate the proportional expression of each variant relative to total *Nos1* expression within the atria and skeletal muscle. Therefore, despite overall reductions in total *Nos1* mRNA levels in the *mdx* LA and skeletal muscle (*Figure 1D*, *E*, *G*, and *H*), we determined whether any differences exist in the composition of splice variants in the remaining total *Nos1*, which could account for the conserved myocardial NOS activity. For all five splice variants, there were no changes in the proportion of Nos1 expression at the transcript level between WT and *mdx* mice, in either the LA or RA (see Supplementary material online, *Figure S8B*–*K*). In contrast, the relative proportion of Nos1-α transcript was increased in the mdx skeletal muscle, with a concomitant reduction in the mRNA expression level of both Nos1-µ and Nos1–2 (see Supplementary material online, *Figure S8L*–*N*). The proportion of Nos1-γ or Nos1-β mRNA expression did not differ significantly between genotypes, although the latter was marginally higher (*P* = 0.0643) in *mdx* mice (see Supplementary material online, *Figure S8O* and *P*). Further, we evaluated whether changes in the expression of NOS1 or NOS3 binding partners could play a role in the maintenance of NOS activity in the *mdx* heart. While Cav-3, Cx43, NOS1AP, and Hsp90 were increased in the *mdx* skeletal muscle compared with WT (see Supplementary material online, *Figure S9A*, *D*, *G*, and *J*), there was no change in the content of these proteins in the atria of *mdx* mice (see Supplementary material online, *Figure S9B*, *E*, *H*, and *K*). Likewise, with the exception of a reduction in Cav-3 and an increase in NOS1AP in the *mdx* RV, there were no significant differences in the expression of these NOS binding partners in the ventricles (see Supplementary material online, *Figure S9F*, *I*, and *L*). We also determined the protein expression of caveolin-1, which binds to and inhibits both NOS1 and NOS3^19,20^; thus, its reduction could lead to an increase in constitutive NOS activity. In the atria, caveolin-1 content was validated using two different caveolin-1 antibodies, both of which were directed against the C-terminal region of caveolin-1 and therefore detected both the alpha and beta isoforms. Caveolin-1 content was lower in the RA than the LA of WT mice and significantly reduced in LA of the *mdx* mice (observed with both antibodies; *Figure 6B* and see Supplementary material online, *Figure S10*). In contrast, caveolin-1 was unchanged in the skeletal muscle and ventricles of *mdx* mice (*Figure 6A* and *C*).

In summary, a higher NOS3-ser^1177^ phosphorylated fraction (in the RA *vs.* LA of both genotypes and in *mdx* RA vs. WT RA) coupled with a reduction in caveolin-1 (in RA vs. LA in both genotypes and in the *mdx* LA *vs.* WT LA) may contribute, at least in part, to equalizing atrial NOS activity between atria and preserving it in the *mdx*, despite significant differences in NOS1 protein content.

## 4. Discussion

We have previously reported that up-regulation of miR-31 in the atrial myocardium of patients and goats with AF leads to translational repression of DYS and faster decay of NOS1 mRNA and demonstrated that the resulting reduction in NOS1 activity contributes to the atrial electrical remodelling that sustains the arrhythmia.^7^ However, patients with DMD, who would be expected to have both atrial dystrophin and NOS1 depletion and who, at least in the skeletal muscle, have been reported to have an up-regulation of miR-31,^12^ are not at a higher risk of developing atrial arrhythmia.^8^ We compared NOS1 expression, protein level, and activity in each of the heart chambers in young *mdx* mice and their WT littermates. Our findings show a significant reduction in NOS1 protein content only in the LA and LV but, surprisingly, and at difference with the skeletal muscle, there were no differences in atrial or ventricular NOS activity between genotypes. Likewise, whereas we confirmed that miR-31 expression was 130-fold higher in the skeletal muscle of *mdx* mice compared with their WT littermates, there was no evidence of miR-31 up-regulation in the *mdx* myocardium. Finally, and in line with data from patients with DMD,^8^ young *mdx* mice did not present ECG abnormalities or increased propensity to develop atrial tachyarrhythmias at a stage where the cardiomyopathic phenotype had not yet developed.

These findings raise a number of questions. First, why is miR-31 up-regulation limited to the *mdx* skeletal muscle and, in its absence, what is causing the reduction in *Nos1* mRNA and protein in the *mdx* hearts? miR-31 controls late muscle differentiation by inhibiting dystrophin translation and is up-regulated in dystrophic skeletal muscles.^12^ The discrepancy between skeletal and myocardial miR-31 expression is unclear and poses the question of the potential cellular sources of miR-31 in human AF. In dystrophic skeletal muscle, miR-31 has preferential localization to satellite cells,^12^ where miR-31 directly contributes to the signalling pathways involved in satellite cell proliferation. Further, *in situ* hybridization staining of rejected cardiac allografts revealed preferential staining of miR-31 to interstitial cells,^21^ postulating the possibility that other atrial cellular sources, e.g. immune cells,^22^ may be responsible for the increased expression of miR-31 in the presence of persistent AF.

Despite reduced NOS1 protein in the *mdx* myocardium, we do not observe a compensatory increase in mRNA expression; in contrast, we see a reduction in *Nos1* LA transcript level. It should be noted that we used two different TaqMan probes: one that targeted *Nos1* exons 26–27 and amplified *all Nos1* splice variants and one that targeted exons 1–2 and amplified only the *Nos1* splice variants that contain the PDZ domain, which is required for anchoring NOS1 protein to the dystrophin complex. Whereas total atrial *Nos1* transcript levels differed significantly between sides but not between genotypes, the *Nos1* splice variants encoding the PDZ domain were significantly reduced in the LA of *mdx* mice compared with WT, mirroring the patterns seen at the protein level. Since this loss of *Nos1* mRNA occurs in the absence of an up-regulation of miR-31, other factors may be responsible for regulating *Nos1* transcription in the *mdx* myocardium. Other miRNAs may influence *Nos1* mRNA expression, and it has been reported that the expression of several miRNAs is altered in the *mdx* mouse and that the expression levels of some of these differ between the skeletal muscle and heart.^23^ Furthermore, more complex regulatory mechanisms are likely to exist, for example, in *mdx* skeletal muscle, NOS1 itself affects miRNA expression through S-nitrosylation of HDAC2, which directly associates with miRNA promoters.^24^

In addition to the reduction in *Nos1* transcript levels, our data indicate that NOS1 depletion in *mdx* mice may arise from breakdown of the dystrophin–glycoprotein complex, destabilization of NOS1 protein, and increased polyubiquitin-mediated degradation of NOS1 by the proteasome. Our previous work in human atrial myocytes from patients with AF showed that preventing binding of miR-31 to dystrophin mRNA not only restored dystrophin protein content but partially recovered NOS1, in the absence of changes in *NOS1* mRNA,^7^ indicating that dystrophin affects NOS1 protein stability as well as its subcellular localization. *In vitro* studies have reported that NOS1 undergoes proteasomal degradation, via ubiquitination of its calmodulin-binding site.^25^ As polyubiquitination preferentially occurs on the inactive, monomeric form of enzyme, this may constitute an important mechanism for removing unstable or nonfunctional NOS1 protein.^25^

Secondly, why—in contrast with our previous findings in the fibrillating human atrial myocardium—was NOS1 depletion in the left chambers of the *mdx* mouse not associated with a reduction in total NOS activity? In line with our findings, Bia *et al.* also reported preservation of total myocardial NOS activity in young *mdx* mice in the absence of significant up-regulation of other NOS isoforms.^16^ Whereas NOS3 Thr495 is a negative regulatory site, and its phosphorylation is associated with a reduction in enzyme activity, Ser1177 phosphorylation stimulates the flux of electrons within the reductase domain and activates NOS3 by increasing the calcium sensitivity of the enzyme.^26^ The increase in NOS3 phosphorylation at this site in the RA myocardium of both genotypes and in the RA of mdx vs. WT could, at least in part, explain why the difference in NOS1 protein level between atrial chambers and genotypes was not accompanied by a concordant variation in total NOS activity. Furthermore, the activity of NOS1 and NOS3 is inhibited by caveolin-1,^19,20^ with studies in mice showing that a loss of caveolin-1 increases NOS3 activity.^27^ Therefore, our findings of a reduction in caveolin-1 protein expression in the *mdx* LA suggest that loss of NOS inhibition by caveolin-1 may also contribute to the conserved NOS activity in the *mdx* atria. Further investigation into the mechanisms contributing to maintain NOS activity in the *mdx* myocardium could include determining genotype differences in the abundance of NOS co-factors; in particular, expression and function of L-arginine transporters have been reported to be significantly increased in the *mdx* myocardium.^28^

While the other known binding partners of NOS1 and NOS3 were unchanged in the *mdx* atria, they were all up-regulated in the *mdx* skeletal muscle, as reported previously,^29,30^ indicating further tissue differences within the *mdx* mice. In particular, our findings indicate that in young *mdx* mice the biochemical phenotype in the skeletal muscle differs from that observed in the myocardium, in keeping with data from patients with DMD indicating a discrepancy between time course with severity of skeletal muscle phenotype and cardiac involvement.^1^

### 4.1 Limitations

Induction of atrial tachyarrhythmias by burst pacing in mice can only offer partial insights on the impact of a genetically modified atrial substrate on the natural history of AF, given the short duration of the arrhythmic episodes. Nevertheless, we have previously shown that disruption of NO signalling is similarly associated with proarrhythmic atrial electrical changes in mice and humans and with greater propensity to AF induction in response to transoesophageal burst pacing in nNOS^−/−^ mice.^7^

We elected to study *mdx* mice at 12–13 weeks of age since the primary aim was to determine whether AF developed as a result of a reduced myocardial NO synthesis, prior to the development of overt cardiac dysfunction and remodelling, as a cardiomyopathic phenotype of any aetiology would be expected to result in an increase in AF susceptibility. In contrast, we showed that the *mdx* myocardium, at difference with the skeletal muscle, does not exhibit an up-regulation of miR-31, a reduction in NO synthesis, or an increase propensity for atrial arrhythmias. Compared with the skeletal muscle, the milder biochemical phenotype of the *mdx* myocardium is in keeping with the low prevalence of AF in patients with DMD and may contribute to explain the delayed development of a cardiomyopathic phenotype in this condition. While we are not suggesting that NO is the sole determinant of the DMD phenotype, reduced NO synthesis leads to muscle ischaemia during exercise and accelerates the dystrophic process in patients with DMD.^4–6^

Investigating the mechanisms underlying differences in NOS1 and caveolin-1 protein content and NOS3 serine 1177 phosphorylation between the left and right heart chambers was outside the scope of this work; however, these findings are not surprising if one considers the variance in the expression of many other proteins between sides as well as the differences in loading conditions between the left and right heart. At least in the skeletal muscle, nNOS expression and protein content are regulated by load and removal of weight bearing from rat hindlimb muscles results in a rapid significant reduction in NOS1 protein and mRNA.^31^

We investigated the effects of dystrophin depletion on NOS1 protein expression and localization using an antibody targeted to the PDZ domain of NOS1 and, thus, focused solely on changes in some NOS1 variants (NOS1-α, -μ, and -2). We cannot, therefore, establish whether compensatory increases in the protein or activity of other NOS1 splice variants (i.e. NOS1-β and -γ) might explain the unaltered total NOS activity or the lack of AF phenotype, although their relatively low expression levels would suggest this is likely not the case.

## 5. Conclusions

In summary, our findings indicate that dystrophin depletion in the *mdx* mouse is associated with a moderate reduction in atrial NOS1 protein content in the absence of changes in total NOS activity, miR-31 expression, or increased AF susceptibility. Compensatory changes in NOS3 phosphorylation and caveolin-1 content may account for the biochemical phenotype of young *mdx* atria compared with skeletal muscle and explain why patients with DMD do not exhibit a higher prevalence of atrial arrhythmias despite a reduction in myocardial NOS1 content.

## Supplementary Material

cvae022_Supplementary_Data

## Data Availability

The data underlying this article will be shared on reasonable request to the corresponding author.

## References

[1] McNally EM, Kaltman JR, Benson DW, Canter CE, Cripe LH, Duan D, Finder JD, Groh WJ, Hoffman EP, Judge DP, Kertesz N, Kinnett K, Kirsch R, Metzger JM, Pearson GD, Rafael-Fortney JA, Raman SV, Spurney CF, Targum SL, Wagner KR, Markham LW. Contemporary cardiac issues in Duchenne muscular dystrophy. Working Group of the National Heart, Lung, and Blood Institute in collaboration with Parent Project Muscular Dystrophy. Circulation 2015;131:1590–1598. 25940966 10.1161/CIRCULATIONAHA.114.015151 PMC4573596

[2] Lai Y, Zhao J, Yue Y, Duan D. α2 and α3 helices of dystrophin R16 and R17 frame a microdomain in the α1 helix of dystrophin R17 for neuronal NOS binding. Proc Natl Acad Sci U S A 2013;110:525–530. 23185009 10.1073/pnas.1211431109 PMC3545791

[3] Brenman JE, Chao DS, Xia H, Aldape K, Bredt DS. Nitric oxide synthase complexed with dystrophin and absent from skeletal muscle sarcolemma in Duchenne muscular dystrophy. Cell 1995;82:743–752. 7545544 10.1016/0092-8674(95)90471-9

[4] Sander M, Chavoshan B, Harris SA, Iannaccone ST, Stull JT, Thomas GD, Victor RG. Functional muscle ischemia in neuronal nitric oxide synthase-deficient skeletal muscle of children with Duchenne muscular dystrophy. Proc Natl Acad Sci U S A 2000;97:13818–13823. 11087833 10.1073/pnas.250379497 PMC17659

[5] Percival JM, Anderson KNE, Huang P, Adams ME, Froehner SC. Golgi and sarcolemmal neuronal NOS differentially regulate contraction-induced fatigue and vasoconstriction in exercising mouse skeletal muscle. J Clin Invest 2010;120:816–826. 20124730 10.1172/JCI40736 PMC2827958

[6] Rebolledo DL, Kim MJ, Whitehead NP, Adams ME, Froehner SC. Sarcolemmal targeting of nNOSμ improves contractile function of mdx muscle. Hum Mol Genet 2016;25:158–166. 26604149 10.1093/hmg/ddv466 PMC4690500

[7] Reilly SN, Liu X, Carnicer R, Recalde A, Muszkiewicz A, Jayaram R, Carena MC, Wijesurendra R, Stefanini M, Surdo NC, Lomas O, Ratnatunga C, Sayeed R, Krasopoulos G, Rajakumar T, Bueno-Orovio A, Verheule S, Fulga TA, Rodriguez B, Schotten U, Casadei B. Up-regulation of miR-31 in human atrial fibrillation begets the arrhythmia by depleting dystrophin and neuronal nitric oxide synthase. Sci Transl Med 2016;8:340ra374. 10.1126/scitranslmed.aac4296 PMC4993239 27225184

[8] Groh WJ, Bhakta D, Tomaselli GF, Aleong RG, Teixeira RA, Amato A, Asirvatham SJ, Cha YM, Corrado D, Duboc D, Goldberger ZD, Horie M, Hornyak JE, Jefferies JL, Kääb S, Kalman JM, Kertesz N, Lakdawala NK, Lambiase PD, Lubitz SA, McMillan HJ, McNally EM, Milone M, Namboodiri N, Nazarian S, Patton KK, Russo V, Sacher F, Santangeli P, Shen W-K, Sobral Filho DC, Stambler BS, Stöllberger C, Wahbi K, Wehrens XHT, Weiner MM, Wheeler MT, Zeppenfeld K. 2022 HRS expert consensus statement on evaluation and management of arrhythmic risk in neuromuscular disorders. Heart Rhythm 2022;19:e61–e120. 35500790 10.1016/j.hrthm.2022.04.022

[9] Shin JH, Hakim CH, Zhang K, Duan D. Genotyping mdx, mdx3cv, and mdx4cv mice by primer competition polymerase chain reaction. Muscle Nerve 2011;43:283–286. 21254096 10.1002/mus.21873 PMC3051167

[10] Zhang YH, Zhang MH, Sears CE, Emanuel K, Redwood C, El-Armouche A, Kranias EG, Casadei B. Reduced phospholamban phosphorylation is associated with impaired relaxation in left ventricular myocytes from neuronal NO synthase-deficient mice. Circ Res 2008;102:242–249. 18007024 10.1161/CIRCRESAHA.107.164798

[11] de Bono JP, Warrick N, Bendall JK, Channon KM, Alp NJ. Radiochemical HPLC detection of arginine metabolism: measurement of nitric oxide synthesis and arginase activity in vascular tissue. Nitric Oxide 2007;16:1–9. 16647284 10.1016/j.niox.2006.03.008

[12] Cacchiarelli D, Incitti T, Martone J, Cesana M, Cazzella V, Santini T, Sthandier O, Bozzoni I. miR-31 modulates dystrophin expression: new implications for Duchenne muscular dystrophy therapy. EMBO Rep 2011;12:136–141. 21212803 10.1038/embor.2010.208 PMC3049433

[13] Kameya S, Miyagoe Y, Nonaka I, Ikemoto T, Endo M, Hanaoka K, Nabeshima Y, Takeda S. alpha1-syntrophin gene disruption results in the absence of neuronal-type nitric-oxide synthase at the sarcolemma but does not induce muscle degeneration. *J Biol Chem* 1999;**274**:2193–2200. 10.1074/jbc.274.4.2193 9890982

[14] Ohlendieck K, Campbell KP. Dystrophin-associated proteins are greatly reduced in skeletal muscle from mdx mice. J Cell Biol 1991;115:1685–1694. 1757468 10.1083/jcb.115.6.1685 PMC2289197

[15] Quinlan JG, Hahn HS, Wong BL, Lorenz JN, Wenisch AS, Levin LS. Evolution of the mdx mouse cardiomyopathy: physiological and morphological findings. Neuromuscul Disord 2004;14:491–496. 15336690 10.1016/j.nmd.2004.04.007

[16] Bia BL, Cassidy PJ, Young ME, Rafael JA, Leighton B, Davies KE, Radda GK, Clarke K. Decreased myocardial nNOS, increased iNOS and abnormal ECGs in mouse models of Duchenne muscular dystrophy. J Mol Cell Cardiol 1999;31:1857–1862. 10525423 10.1006/jmcc.1999.1018

[17] Fleming I, Fisslthaler B, Dimmeler S, Kemp BE, Busse R. Phosphorylation of Thr495 regulates Ca2+/calmodulin-dependent endothelial nitric oxide synthase activity. Circ Res 2001;88:68e–75. 10.1161/hh1101.092677 11397791

[18] Balke JE, Zhang L, Percival JM. Neuronal nitric oxide synthase (nNOS) splice variant function: insights into nitric oxide signaling from skeletal muscle. Nitric Oxide 2019;82:35–47. 30503614 10.1016/j.niox.2018.11.004

[19] Sato Y, Sagami I, Shimizu T. Identification of caveolin-1-interacting sites in neuronal nitric-oxide synthase. Molecular mechanism for inhibition of NO formation. J Biol Chem 2004;279:8827–8836. 14681230 10.1074/jbc.M310327200

[20] Ju H, Zou R, Venema VJ, Venema RC. Direct interaction of endothelial nitric-oxide synthase and caveolin-1 inhibits synthase activity. J Biol Chem 1997;272:18522–18525. 9228013 10.1074/jbc.272.30.18522

[21] Duong Van Huyen JP, Tible M, Gay A, Guillemain R, Aubert O, Varnous S, Iserin F, Rouvier P, François A, Vernerey D, Loyer X, Leprince P, Empana J-P, Bruneval P, Loupy A, Jouven X. MicroRNAs as non-invasive biomarkers of heart transplant rejection. Eur Heart J 2014;35:3194–3202. 25176944 10.1093/eurheartj/ehu346

[22] Ripamonti A, Provasi E, Lorenzo M, De Simone M, Ranzani V, Vangelisti S, Curti S, Bonnal RJP, Pignataro L, Torretta S, Geginat J, Rossetti G, Pagani M, Abrignani S. Repression of miR-31 by BCL6 stabilizes the helper function of human follicular helper T cells. Proc Natl Acad Sci U S A 2017;114:12797–12802. 29133396 10.1073/pnas.1705364114 PMC5715737

[23] Roberts TC, Blomberg KEM, McClorey G, El Andaloussi S, Godfrey C, Betts C, Coursindel T, Gait MJ, Smith CI, Wood MJ. Expression analysis in multiple muscle groups and serum reveals complexity in the microRNA transcriptome of the mdx mouse with implications for therapy. Mol Ther Nucleic Acids 2012;1:e39. 23344181 10.1038/mtna.2012.26 PMC3437806

[24] Cacchiarelli D, Martone J, Girardi E, Cesana M, Incitti T, Morlando M, Nicoletti C, Santini T, Sthandier O, Barberi L, Auricchio A, Musarò A, Bozzoni I. MicroRNAs involved in molecular circuitries relevant for the Duchenne muscular dystrophy pathogenesis are controlled by the dystrophin/nNOS pathway. Cell Metab 2010;12:341–351. 20727829 10.1016/j.cmet.2010.07.008

[25] Bender AT, Demady DR, Osawa Y. Ubiquitination of neuronal nitric-oxide synthase in vitro and in vivo. J Biol Chem 2000;275:17407–17411. 10751385 10.1074/jbc.M000155200

[26] Garcia V, Sessa WC. Endothelial NOS: perspective and recent developments. Br J Pharmacol 2019;176:189–196. 30341769 10.1111/bph.14522 PMC6295413

[27] Wunderlich C, Schober K, Lange SA, Drab M, Braun-Dullaeus RC, Kasper M, Schwencke C, Schmeisser A, Strasser RH. Disruption of caveolin-1 leads to enhanced nitrosative stress and severe systolic and diastolic heart failure. Biochem Biophys Res Commun 2006;340:702–708. 16380094 10.1016/j.bbrc.2005.12.058

[28] Ramachandran J, Schneider JS, Crassous PA, Zheng R, Gonzalez JP, Xie LH, Beuve A, Fraidenraich D, Peluffo RD. Nitric oxide signalling pathway in Duchenne muscular dystrophy mice: up-regulation of L-arginine transporters. Biochem J 2013;449:133–142. 23009292 10.1042/BJ20120787 PMC4365916

[29] Vaghy PL, Fang J, Wu W, Vaghy LP. Increased caveolin-3 levels in mdx mouse muscles. FEBS Lett 1998;431:125–127. 9684879 10.1016/s0014-5793(98)00738-8

[30] Ségalat L, Grisoni K, Archer J, Vargas C, Bertrand A, Anderson JE. CAPON expression in skeletal muscle is regulated by position, repair, NOS activity, and dystrophy. Exp Cell Res 2005;302:170–179. 15561099 10.1016/j.yexcr.2004.09.007

[31] Tidball JG, Lavergne E, Lau KS, Spencer MJ, Stull JT, Wehling M. Mechanical loading regulates NOS expression and activity in developing and adult skeletal muscle. Am J Physiol 1998;275:C260–C266. 9688857 10.1152/ajpcell.1998.275.1.C260

