# Supplementary material for: Why is early-onset atrial fibrillation uncommon in patients with Duchenne muscular dystrophy? Insights from the mdx mouse
Source: Cardiovasc Res. 2024 Jan 25;120(5):519–30. doi: 10.1093/cvr/cvae022 (PMC11060487; doi:10.1093/cvr/cvae022)
Supplement: cvae022_Supplementary_Data [file cvae022_supplementary_data.pdf]

Why is early-onset atrial fibrillation uncommon in patients with Duchenne muscular dystrophy? Insights from the mdx mouse.

My-Nhan Nguyen[1#], Charlotte Hooper[1#], Matilde Stefanini[1], Besarte Vrellaku[1], Ricardo Carnicer[1], Matthew J. Wood[2], Jillian N. Simon[1*], Barbara Casadei[1*].

[1]Division of Cardiovascular Medicine, Radcliffe Department of Medicine, John Radcliffe Hospital, University of Oxford, UK.
[2]Department of Paediatrics & Muscular Dystrophy UK Oxford Neuromuscular Centre, University of Oxford, South Parks Road, Oxford, UK.

# these authors contributed equally to the manuscript
* these authors contributed equally to the supervision of this work.

Supplementary Table 1. ECG Parameters in wild-type and *mdx* mice.

	**WT**	***mdx***	***P value***
N	26	25	
Age (weeks)	12.55 ± 0.37	12.66 ± 0.48	*0.25*
P-wave duration (ms)	19.10 ± 1.63	19.28 ± 2.15	*0.73*
PR interval (ms)	38.76 ± 2.67	38.50 ± 2.79	*0.74*
QRS duration (ms)	10.97 ± 1.30	11.10 ± 1.96	*0.79*
QT interval (ms)	60.33 ± 6.00	58.95 ± 8.58	*0.51*
QTc interval (ms)	49.45± 4.61	50.04 ± 5.75	*0.70*
Heart rate (bpm)	406 ± 51.58	438 ± 48.77	*0.03*

Data presented as mean ± SD. P values by Student's unpaired t-test. Abbreviations: bpm, beats per minute; ms, milliseconds

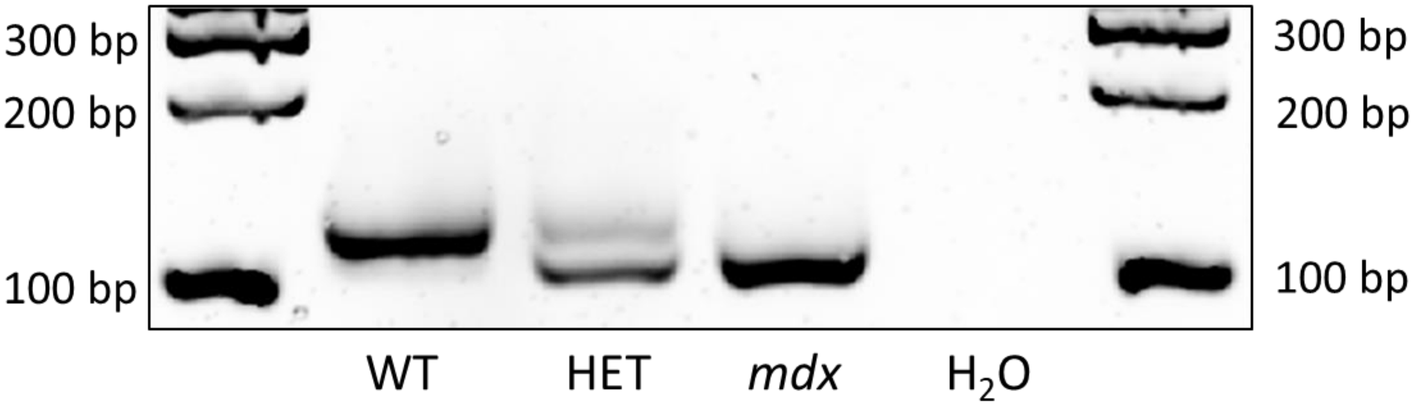

Supplementary Figure 1. Representative genotyping results for wild-type (WT), heterozygous (HET) and male dystrophin-deficient (*mdx*) mice. A sample from a female *mdx* mouse was used to represent heterozygous bands, whilst a water (H_2O) sample without tail DNA was used as negative control (no bands). The expected product sizes for a WT and *mdx* band are 134 and 117 base pairs, respectively.

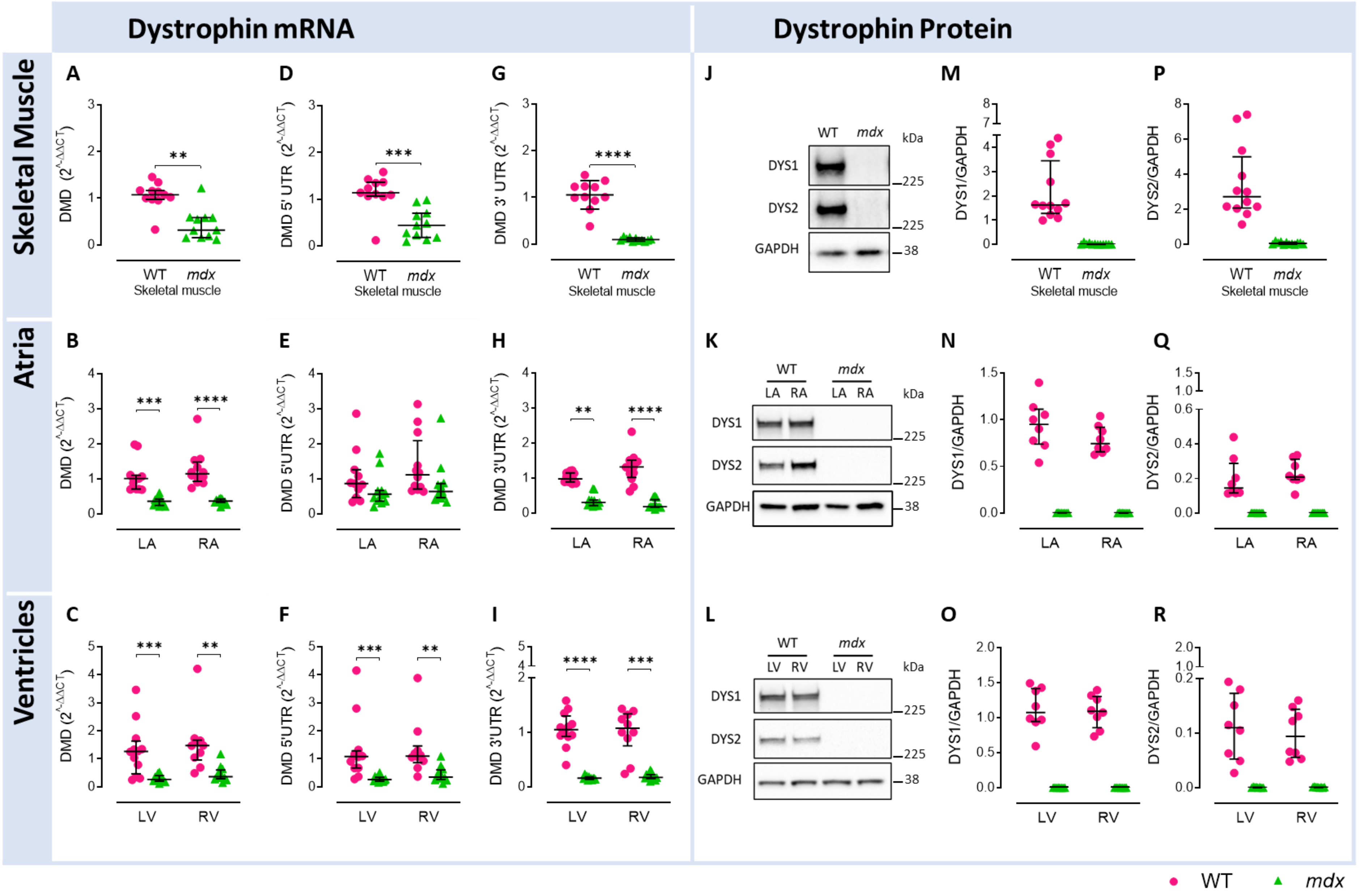

Dystrophin mRNA
Dystrophin Protein
Skeletal Muscle
Atria
Ventricles
A
B
C
D
E
F
G
H
I
J
K
L
M
N
O
P
Q
R
DMD (2^-ΔΔCT)
DMD 5' UTR (2^-ΔΔCT)
DMD 3' UTR (2^-ΔΔCT)
DMD 5'UTR (2^-ΔΔCT)
DMD 3'UTR (2^-ΔΔCT)
WT mdx
Skeletal muscle
LA RA
LV RV
DYS1
DYS2
GAPDH
kDa
225
38
DYS1/GAPDH
DYS2/GAPDH
WT
mdx

Supplementary Figure 2.

Loss of dystrophin at the protein and mRNA level in *mdx* mice. **A.** mRNA expression of *DMD* (i.e. gene encoding dystrophin) from left (LA) and right atria (RA) of wildtype (WT) and *mdx* mice. N = 12 per group. **B.** mRNA expression of *DMD* from left (LV) and right ventricle (RV) of WT and *mdx* mice. N = 10-12 per group. **C.** mRNA expression of *DMD* from skeletal muscle of WT and *mdx* mice. N = 11 per group. **D-I.** Atrial, ventricular and skeletal mRNA expression of dystrophin at 5' untranslated region (UTR) and 3' UTR, which corresponds to N- and C-terminus domains of the dystrophin protein, respectively. N = 8 (**D-E** and **G-H**) and N = 11 (**F** and **I**) per group. **J-L** Representative immunoblots of DYS1, DYS2 and GAPDH in WT and *mdx* atria, ventricles and skeletal muscle. GAPDH was used as housekeeping control for all immunoblots. **M-R.** Densitometry analysis revealing protein content of the rod domain (DYS1) and C-terminus domain (DYS2) of dystrophin from LA and RA, LV and RV, and skeletal muscle protein homogenates of WT and *mdx* mice. N = 8 (**M-N** and **P-Q**) and N = 12 (**O** and **R**) per group. Data are expressed as mean ± SD (**I**) or median ± IQR (**A-H** and **M-R**). P values were determined by Student's unpaired t-test (**I**), Mann-Whitney U test (**C**, **F**, **O** and **R**) or Kruskal-Wallis ANOVA test with Dunn's multiple comparison (**A-B**, **D-E**, **G-H**, **M-N** and **P-Q**). **P<0.01, ***P<0.001, ****P<0.0001.

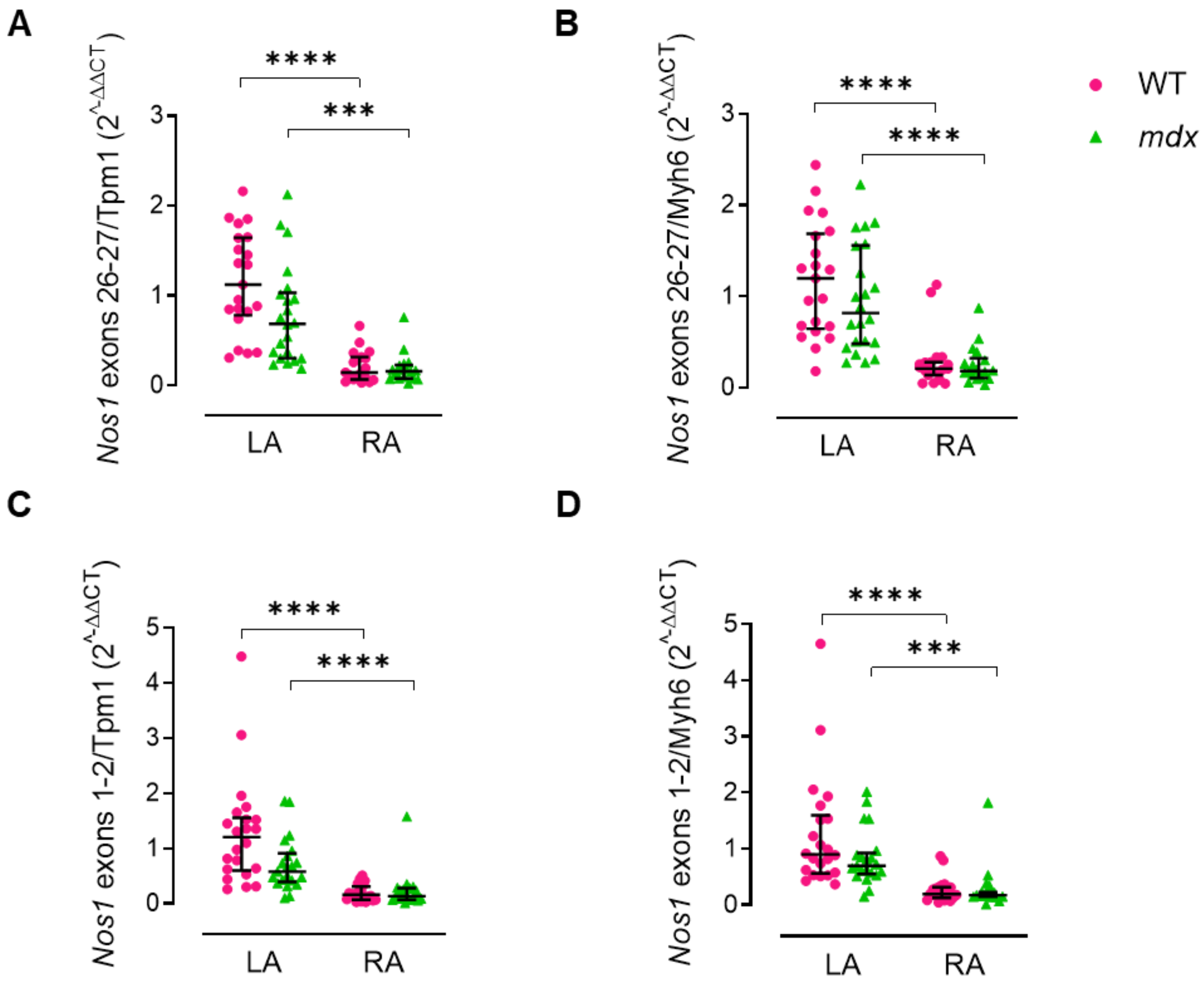

Supplementary Figure 3.

A-D. mRNA expression of total *Nos1* and PDZ domain-containing splice variants of *Nos1* (*Nos1*-α, μ, and 2) normalised to cardiomyocyte markers tropomyosin 1 (*Tpm1*) and myosin heavy chain 6 (*Myh6*) in LA and RA of WT and *mdx* mice; N = 18-22 per group. Data are expressed as median ± IQR. P values were determined by two-way ANOVA with Bonferroni's multiple comparison on log transformed data (**B-C**) or Kruskal-Wallis ANOVA test with Dunn's multiple comparison (**A** and **D**). ***P<0.001, ****P<0.0001.

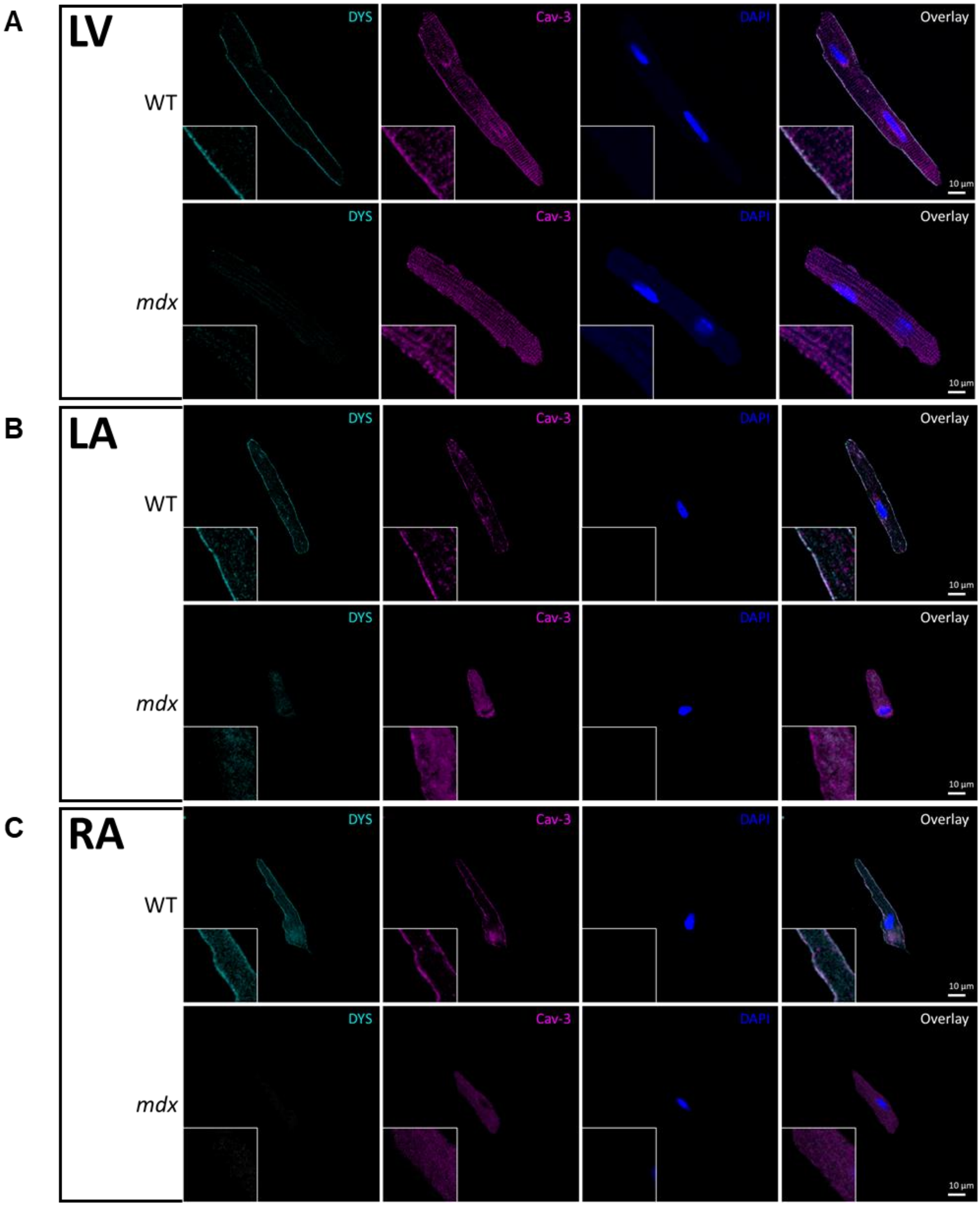

A
LV
WT
mdx
DYS
Cav-3
DAPI
Overlay
10 µm
B
LA
C
RA

Supplementary Figure 4.

A-C. Representative immunofluorescence staining of dystrophin (DYS; cyan), caveolin-3 (Cav-3; magenta), and DAPI (blue) in isolated adult cardiomyocytes from WT and *mdx* LV, LA, and RA. Dystrophin localises to the sarcolemma membrane in WT mice; staining in absent in the *mdx*. Caveolin-3 was used as a sarcolemma membrane maker.

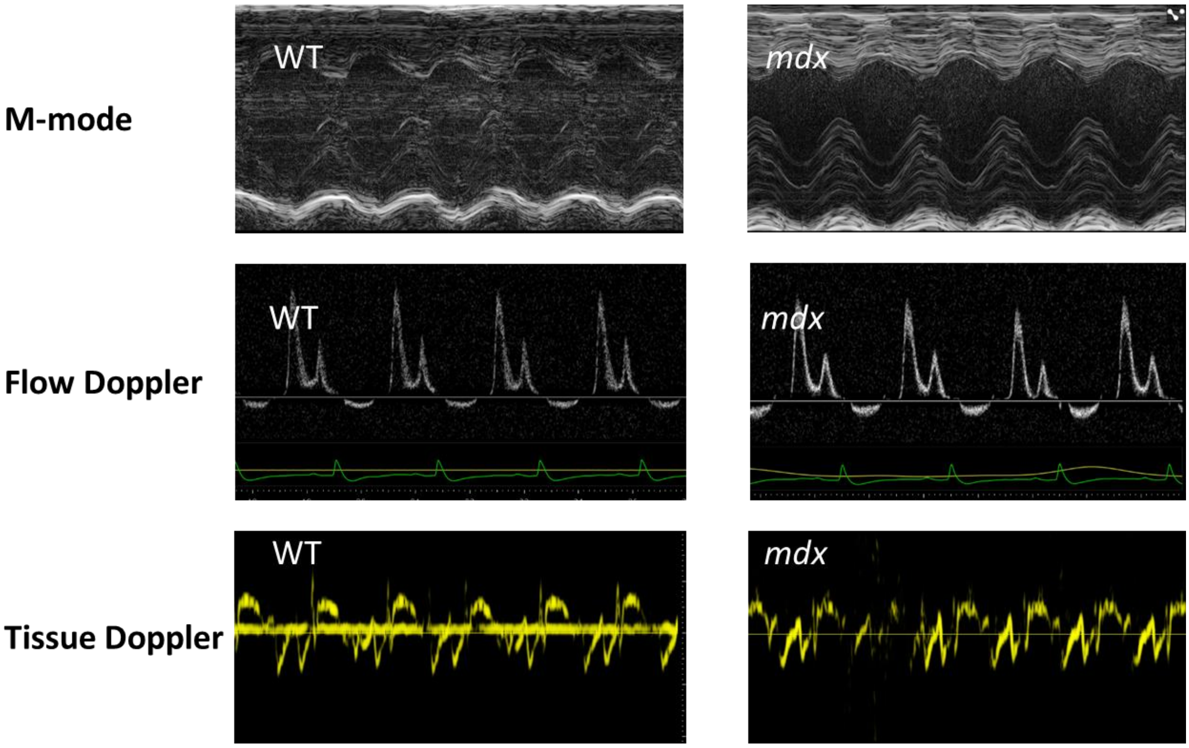

Supplementary Figure 5.

Representative traces of LV M-mode, flow Doppler, and tissue Doppler from WT and *mdx* mice taken during echocardiography.

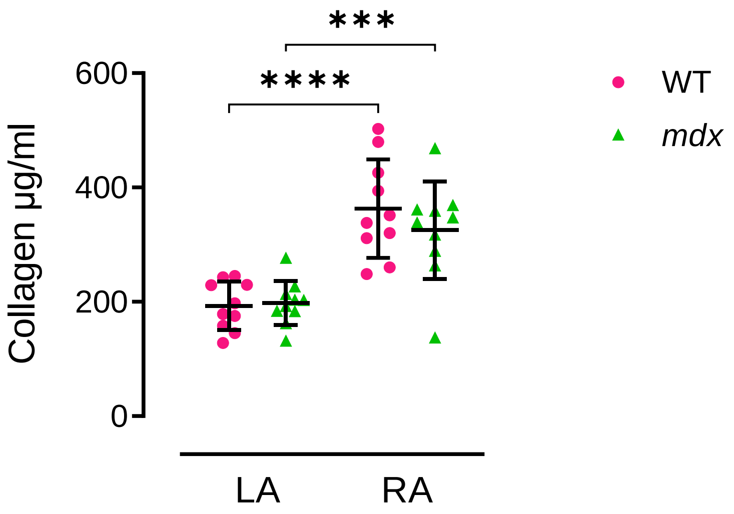

Supplementary Figure 6. Atrial collagen content in WT and *mdx* mice. N = 10 per group. Data are expressed as mean ± SD. P values were determined by Two-way ANOVA with Bonferroni's multiple comparison test. ***P<0.001, ****P<0.0001

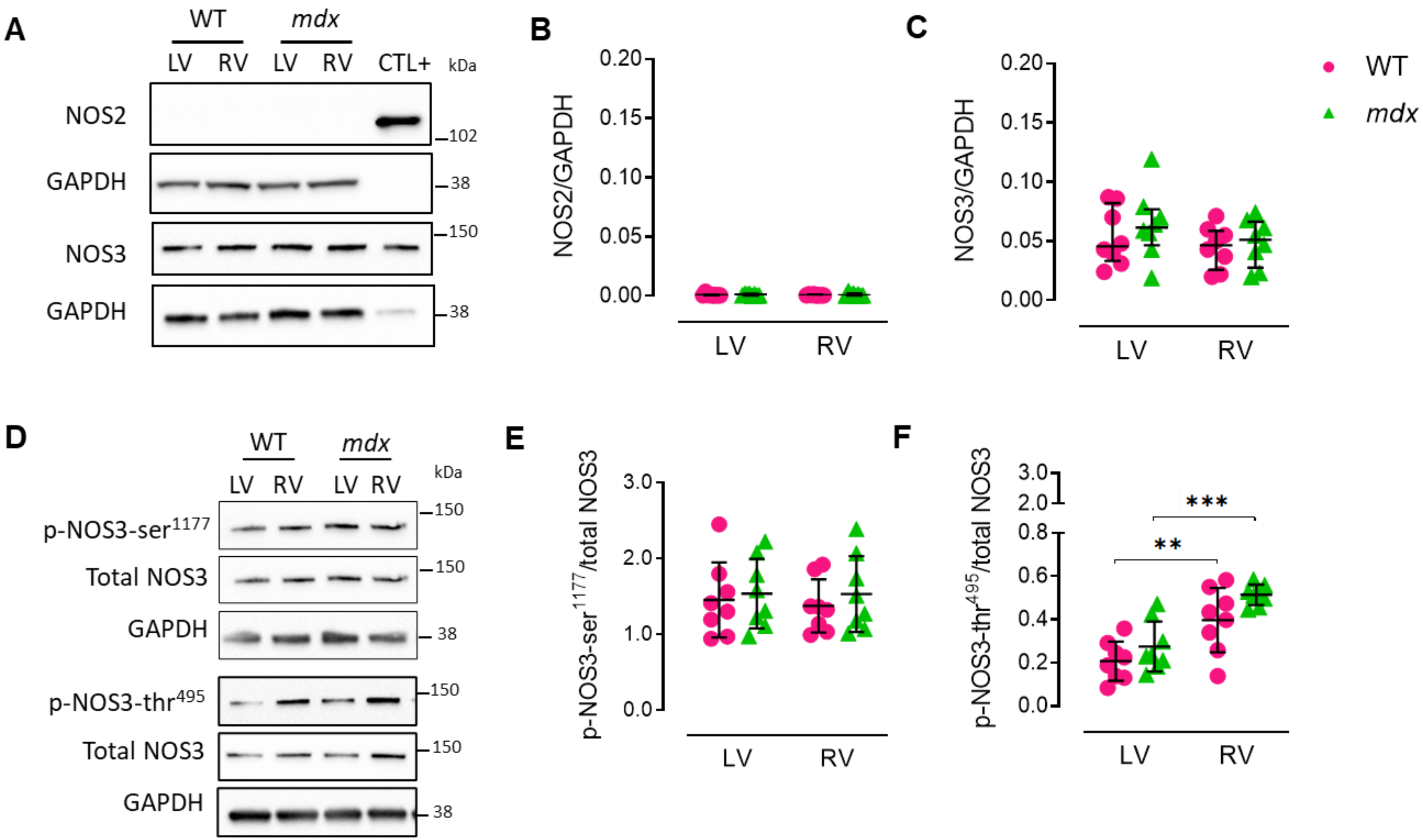

A
WT
mdx
LV RV LV RV CTL+
kDa
NOS2
GAPDH
NOS3
GAPDH
102
38
150
38
B
NOS2/GAPDH
0.20
0.15
0.10
0.05
0.00
LV
RV
C
NOS3/GAPDH
WT
mdx
D
p-NOS3-ser1177
Total NOS3
GAPDH
p-NOS3-thr495
Total NOS3
GAPDH
E
p-NOS3-ser1177/total NOS3
3.0
2.0
1.0
0.0
F
p-NOS3-thr495/total NOS3
0.8
0.6
0.4
0.2
**
***

Supplementary Figure 7. Ventricular protein content and phosphorylation status of other NOS isoforms in *mdx* and WT mice.

A-C. Densitometry analysis and representative immunoblots showing LV and RV protein content of inducible NOS (NOS2) and endothelial NOS (NOS3) in the LV and RV of WT and *mdx* mice. N = 8 per group. Lipopolysaccharide-activated macrophages and WT lung tissue were used as positive controls (+CTL) for NOS2 and NOS3 protein content, respectively. **D-F.** Densitometry analysis and representative immunoblots of phosphorylated NOS3 in ventricular homogenates of WT and *mdx* mice. NOS3 phosphorylation was examined at serine^{1177} (p-NOS3-ser^{1177}) and threonine^{495} (p-NOS3-thr^{495}); N = 8 per group. Data are expressed as mean ± SD (**E-F**) or median ± IQR (**B-C**). P values were determined by two-way ANOVA with Bonferroni's multiple comparison test (**E-F**) or Kruskal-Wallis ANOVA test with Dunn's multiple comparisons (**B-C**). $^{**}P<0.01$, $^{***}P<0.001$.

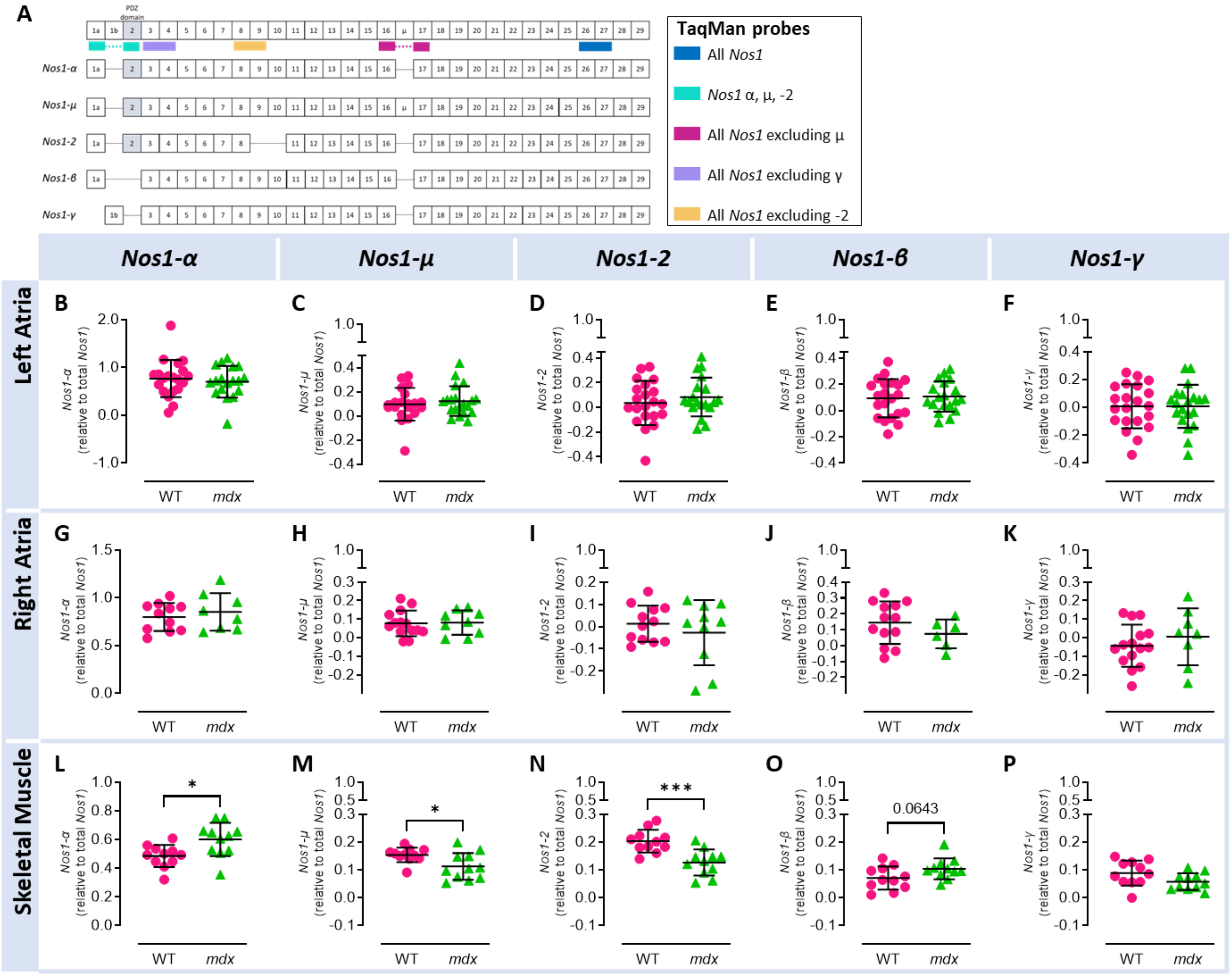

A
PDZ domain
TaqMan probes
All Nos1
Nos1 α, μ, -2
All Nos1 excluding μ
All Nos1 excluding γ
All Nos1 excluding -2
Nos1-α
Nos1-μ
Nos1-2
Nos1-β
Nos1-γ
Left Atria
Right Atria
Skeletal Muscle
B
C
D
E
F
G
H
I
J
K
L
M
N
O
P
(relative to total Nos1)
WT
mdx
*
*
***
0.0643

Supplementary Figure 8. Composition of the *Nos1* splice variants that constitute total *Nos1* mRNA content in the left (LA) and right atria (RA), and skeletal muscle in *mdx* and WT mice. **A.** Exon binding sites of the *Nos1* TaqMan probes (ThermoFisher Scientific) within the five *Nos1* splice variants (*Nos*1 splice variant exon structure diagram based on schematic presented in Balke et al [2019] *Nitric Oxide*)[18]. **B-F**. Left atrial mRNA expression of *Nos*1-α, *Nos*1-μ, *Nos*1-2, *Nos*1-β and *Nos*1-γ each relative to total *Nos*1 transcript level in WT and *mdx* mice. N = 18-20 per group. **G-K**. Right atrial mRNA expression of *Nos1*-α, *Nos1*-μ, *Nos1*-2, *Nos1*-β and *Nos1*-γ each relative to total *Nos1* transcript level in WT and *mdx* mice. N = 6-15 per group. **L-P**. Skeletal muscle mRNA expression of *Nos*1-α, *Nos*1-μ, *Nos*1-2, *Nos1*-β and *Nos1*-γ each relative to total *Nos1* transcript level in WT and *mdx* mice. N = 11 per group. Data are expressed as mean ± SD. P values were determined by Student's unpaired t-test. *P<0.05, ***P<0.001.

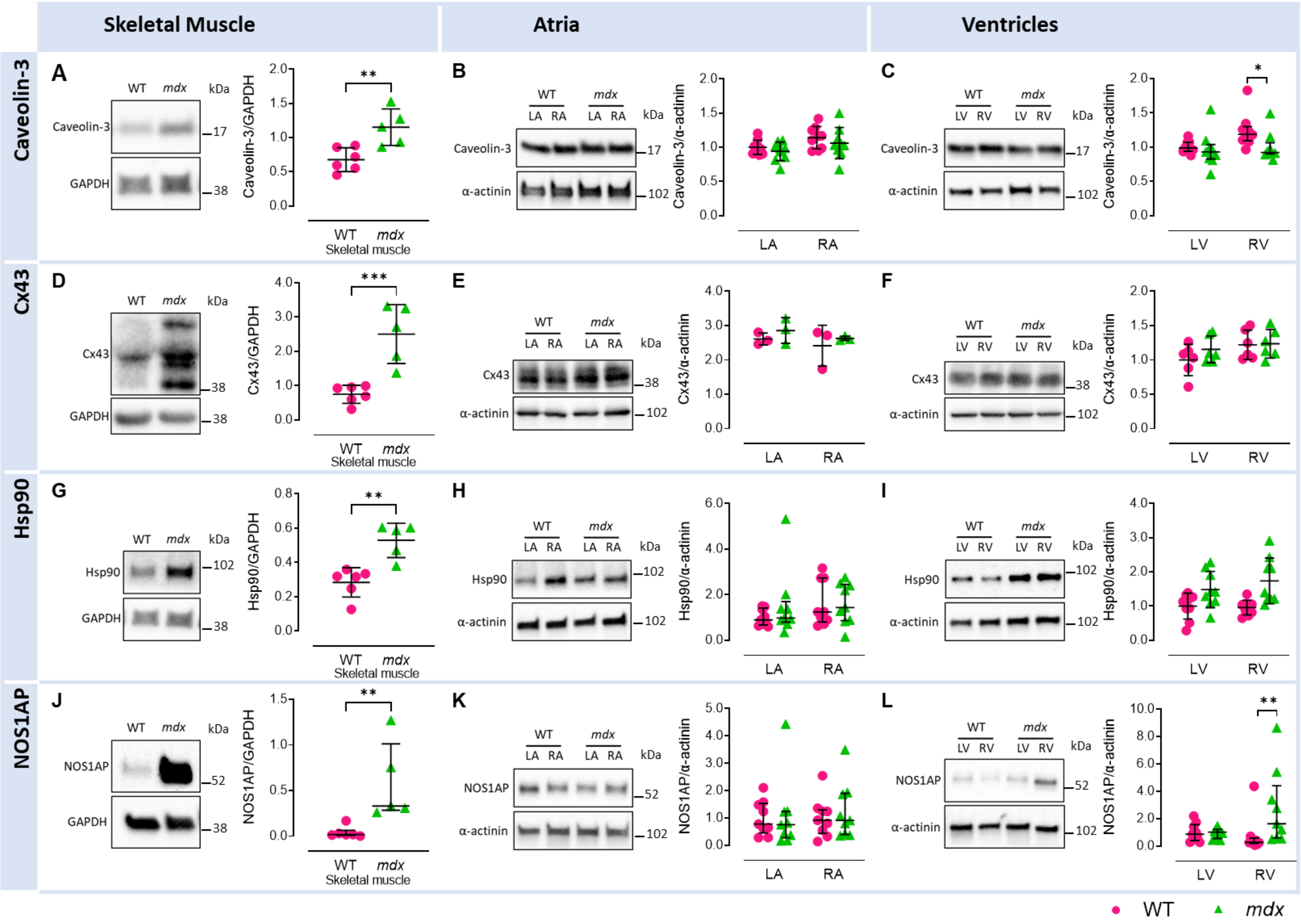
Skeletal Muscle
Atria
Ventricles
Caveolin-3
Cx43
Hsp90
NOS1AP
A
WT mdx kDa
Caveolin-3
17
GAPDH
38
Caveolin-3/GAPDH
**
WT mdx
Skeletal muscle
B
WT mdx
LA RA LA RA
kDa
Caveolin-3
17
α-actinin
102
Caveolin-3/α-actinin
LA RA
C
WT mdx
LV RV LV RV
kDa
Caveolin-3
17
α-actinin
102
Caveolin-3/α-actinin
*
LV RV
D
WT mdx kDa
Cx43
38
GAPDH
38
Cx43/GAPDH
***
WT mdx
Skeletal muscle
E
WT mdx
LA RA LA RA
kDa
Cx43
38
α-actinin
102
Cx43/α-actinin
LA RA
F
WT mdx
LV RV LV RV
kDa
Cx43
38
α-actinin
102
Cx43/α-actinin
LV RV
G
WT mdx kDa
Hsp90
102
GAPDH
38
Hsp90/GAPDH
**
WT mdx
Skeletal muscle
H
WT mdx
LA RA LA RA
kDa
Hsp90
102
α-actinin
102
Hsp90/α-actinin
LA RA
I
WT mdx
LV RV LV RV
kDa
Hsp90
102
α-actinin
102
Hsp90/α-actinin
LV RV
J
WT mdx kDa
NOS1AP
52
GAPDH
38
NOS1AP/GAPDH
**
WT mdx
Skeletal muscle
K
WT mdx
LA RA LA RA
kDa
NOS1AP
52
α-actinin
102
NOS1AP/α-actinin
LA RA
L
WT mdx
LV RV LV RV
kDa
NOS1AP
52
α-actinin
102
NOS1AP/α-actinin
**
LV RV
WT mdx

Supplementary Figure 9.

Protein expression levels of NOS1 and NOS3 binding partners in *mdx* mice. **A-L.** Representative immunoblots and densitometry analysis of caveolin-3 (**A-C**), connexin-43 (Cx43; **D-F**), heat shock protein 90 (Hsp90; **G-I**), NOS1AP (**J-L**) and GAPDH (housekeeping control for skeletal muscle) or α-actinin (housekeeping control for the heart) in WT and *mdx* LA, RA, LV, RV and skeletal muscle. N= 3 (**E**), N = 5-6 (**A, D, F-G** and **J**), and N = 9 (**B-C**, **H-I** and **K-L**) per group. Data are expressed as mean ± SD (**D**, **F**-**G** and **I-K**) or median ± IQR (**A-C**, **E**, **H** and **L**). P values were determined by Student's unpaired t-test (**D**, **G** and **J**), Mann-Whitney U test (**A**), two-way ANOVA with Bonferroni's multiple comparison (**F**, **I** and **K**) on log transformed data (**B-C**) or Kruskal-Wallis ANOVA test with Dunn's multiple comparison (**E**, **H** and **L**). *P<0.05, **P<0.01, ***P<0.001.

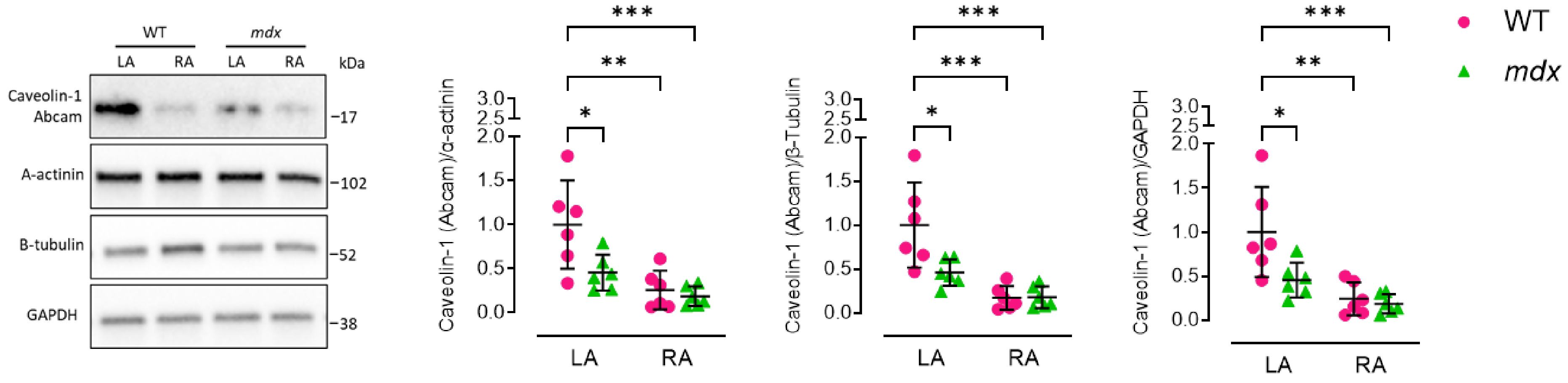

Supplementary Figure 10.

Reduced left atrial (LA) protein expression of caveolin-1 in *mdx* mice, as validated using an alternative caveolin-1 antibody (Abcam). Representative immunoblots and densitometry analysis of caveolin-1 in LA and RA of WT and *mdx* mice. The following housekeeping controls were used, α-actinin, β-tubulin, and GAPDH. N = 6 per group. Data are expressed as mean ± SD. P values were determined by one-way ANOVA with Bonferroni's multiple comparison. *P<0.05, **P<0.01, ***P<0.001.

1J

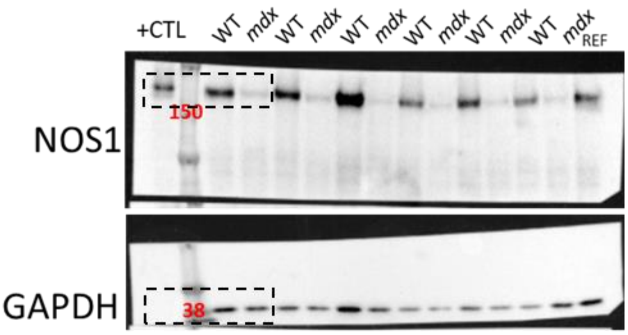

1K

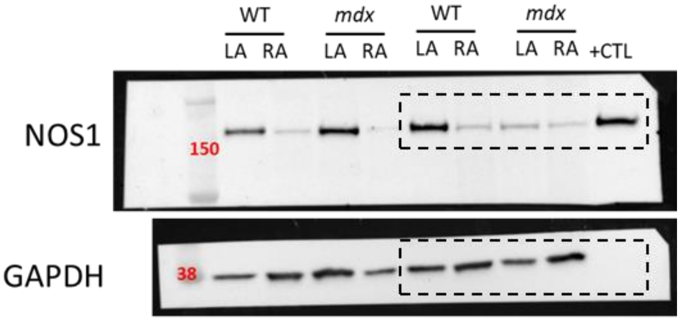

1L

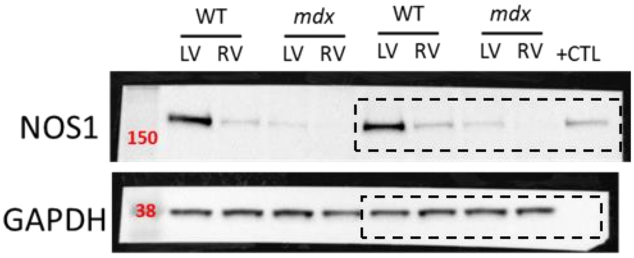

Supplementary Figure 11.

Uncropped membranes for Western blots presented in Figure 1.

2D

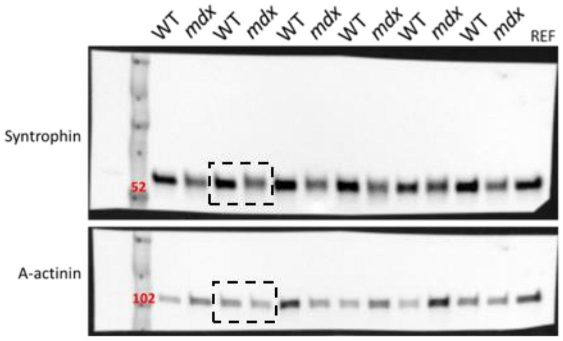

2E

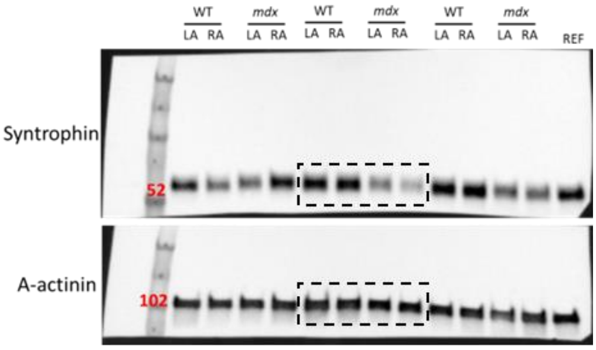

2F

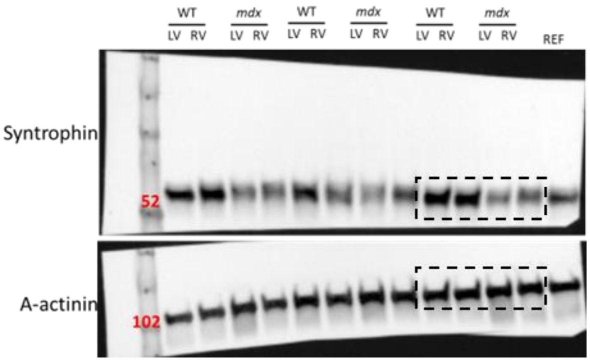

Supplementary Figure 12.

Uncropped membranes for Western blots presented in Figure 2.

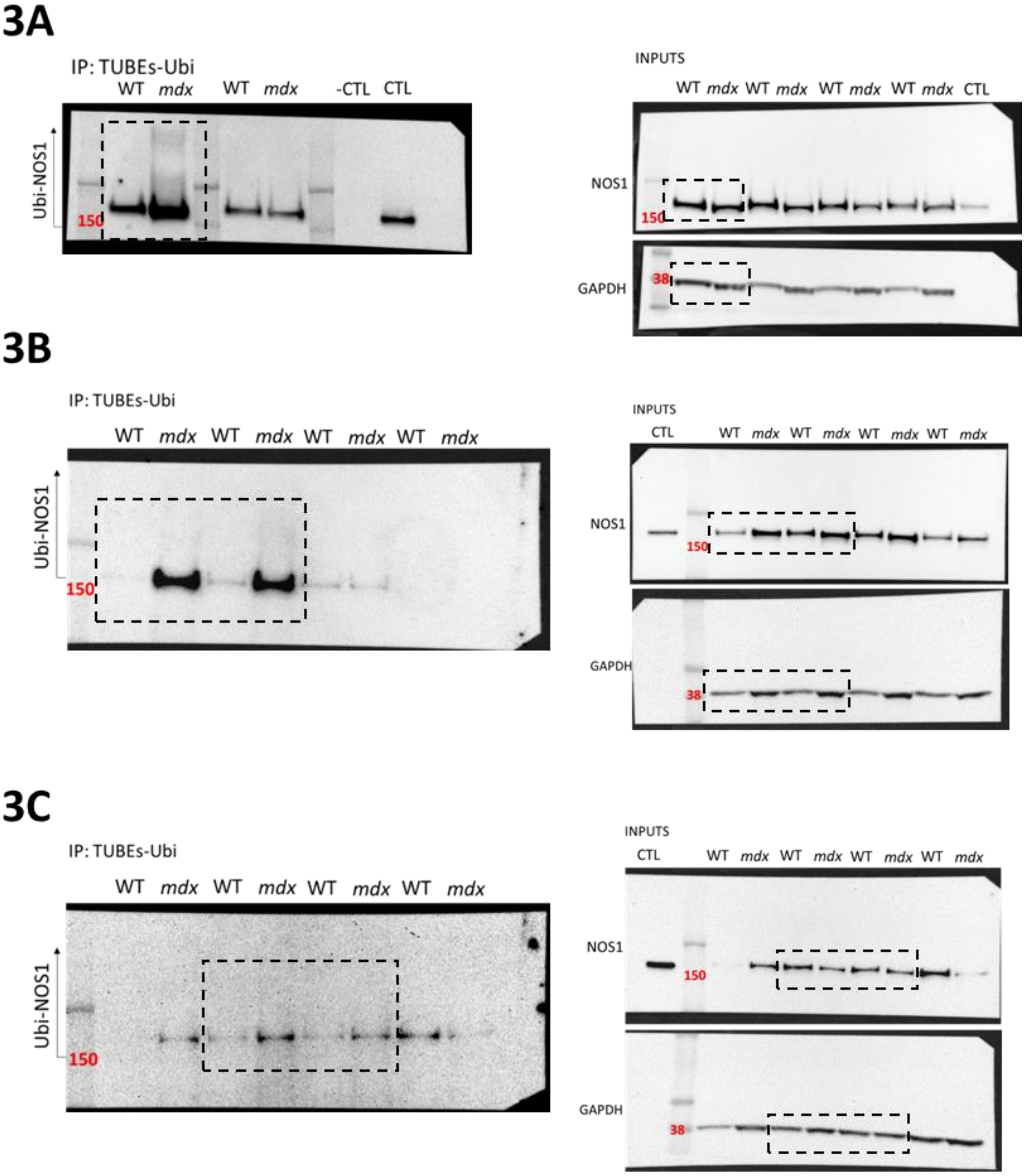

Supplementary Figure 13.

Uncropped membranes for Western blots presented in Figure 3.

5D

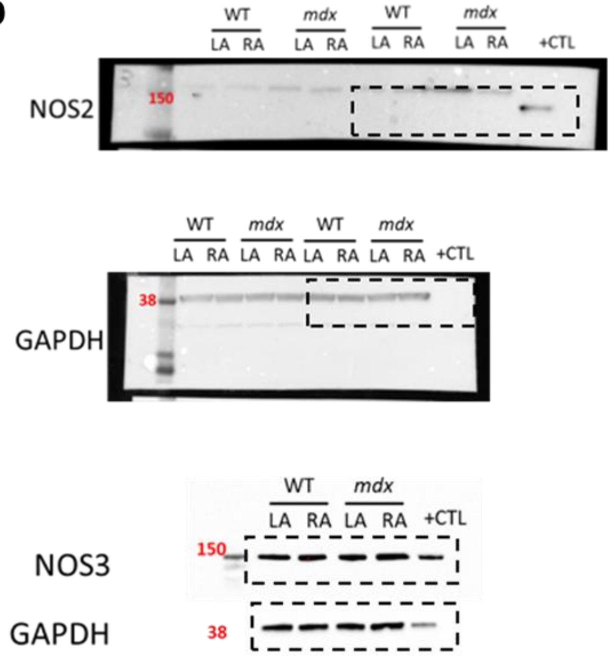

5G

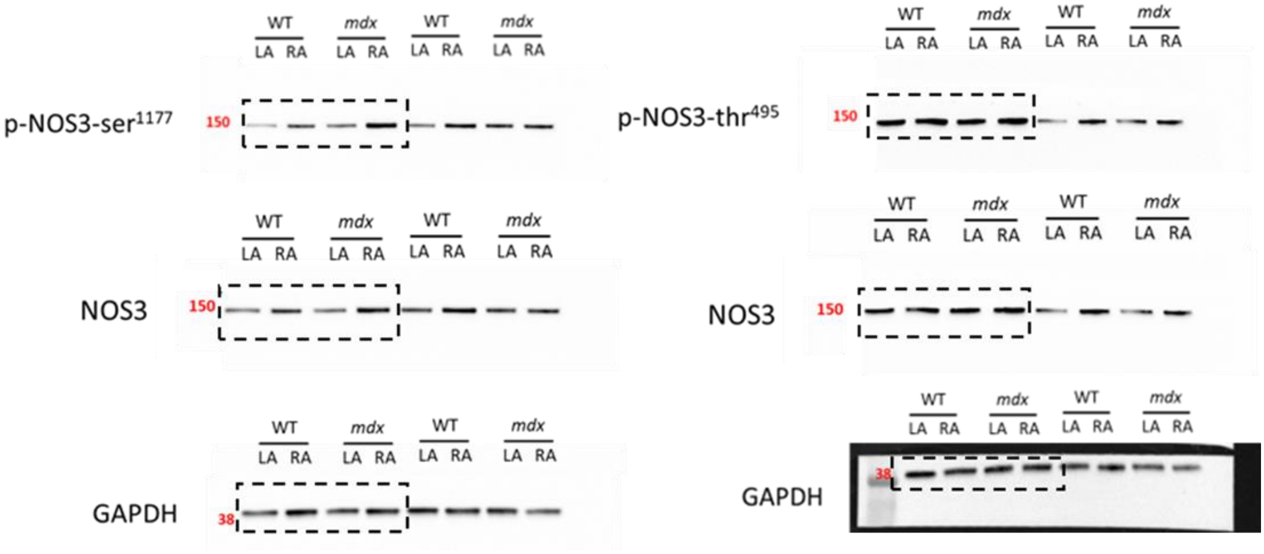

Supplementary Figure 14.

Uncropped membranes for Western blots presented in Figure 5.

6A

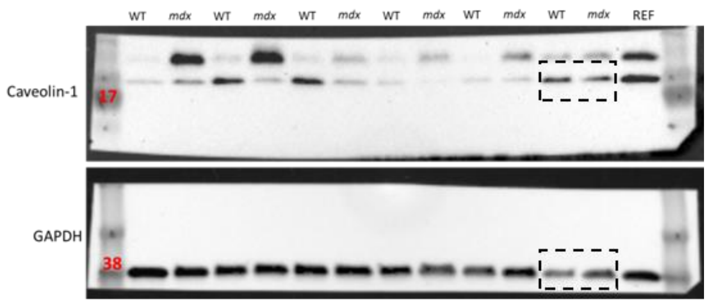

6B

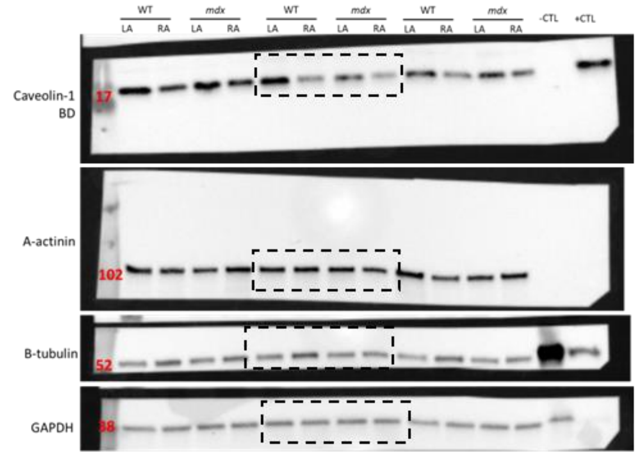

6C

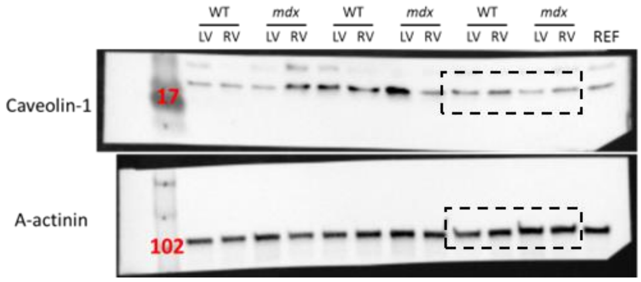

Supplementary Figure 15.

Uncropped membranes for Western blots presented in Figure 6.

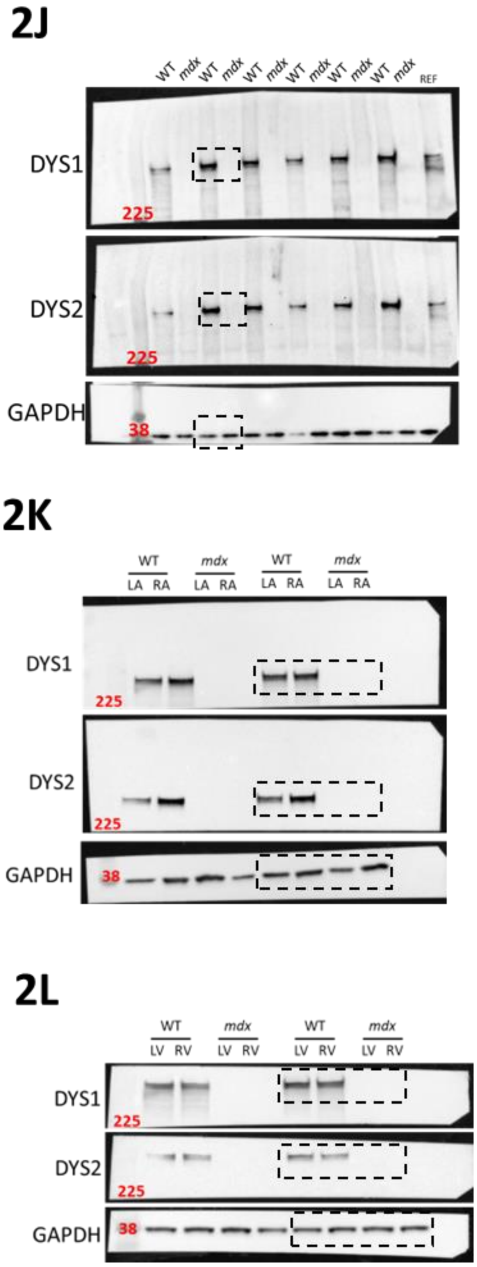

Supplementary Figure 16.

Uncropped membranes for Western blots presented in Supplementary Figure 2.

7A

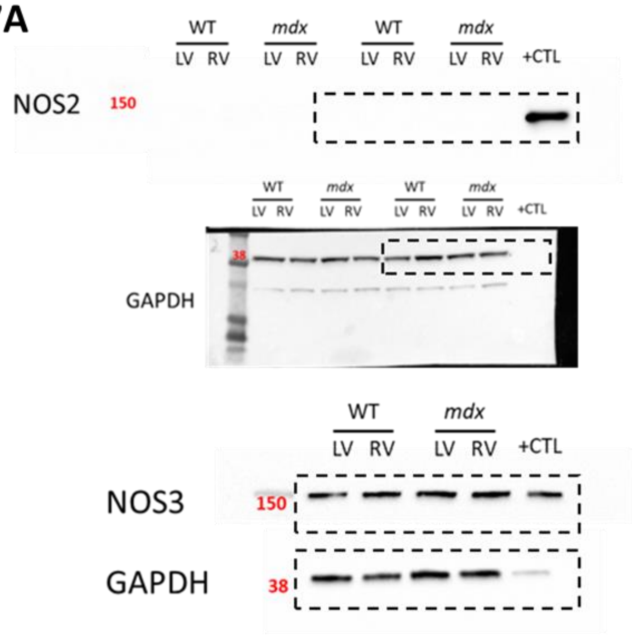

7D

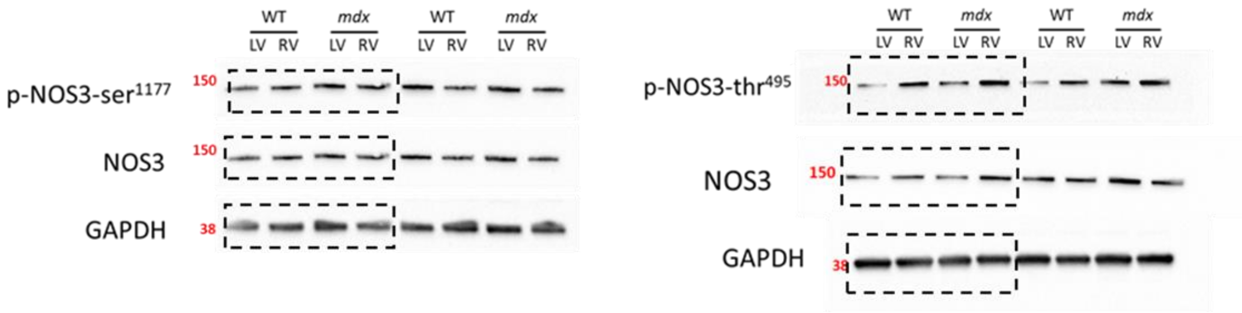

Supplementary Figure 17.

Uncropped membranes for Western blots presented in Supplementary Figure 7.

9A & 9G

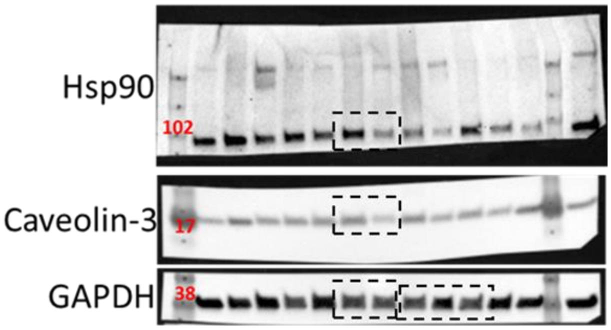

9B

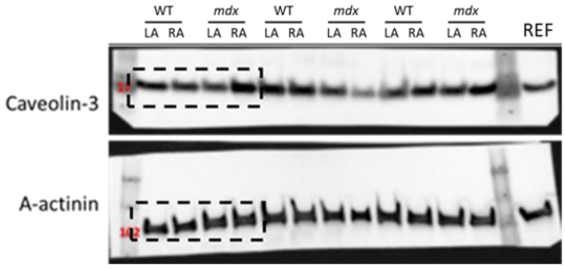

9C

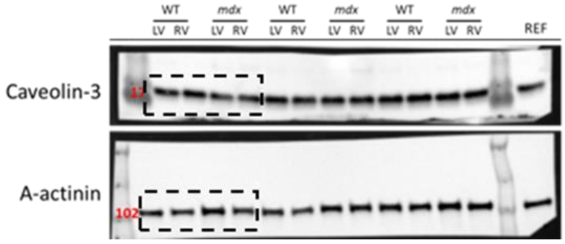

9D

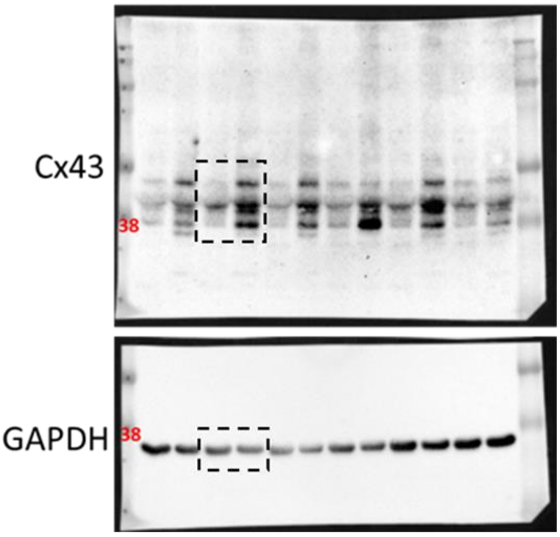

9E

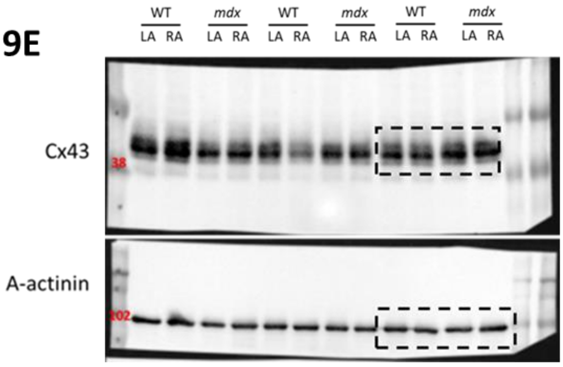

9F

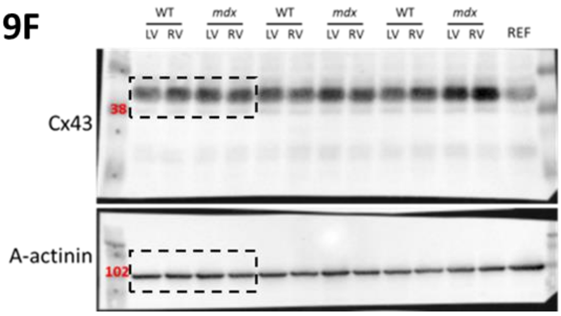

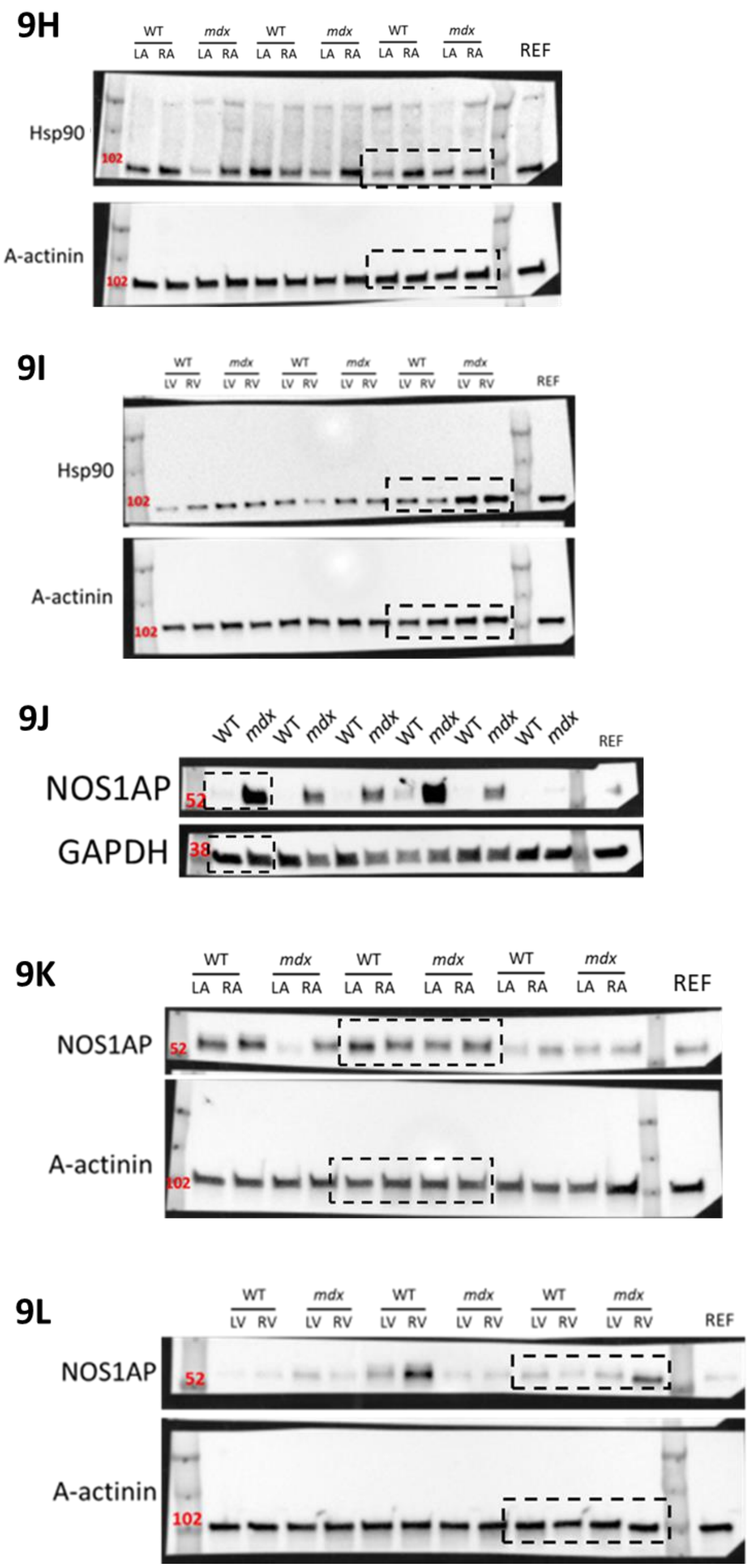

Supplementary Figure 18.

Uncropped membranes for Western blots presented in Supplementary Figure 9.

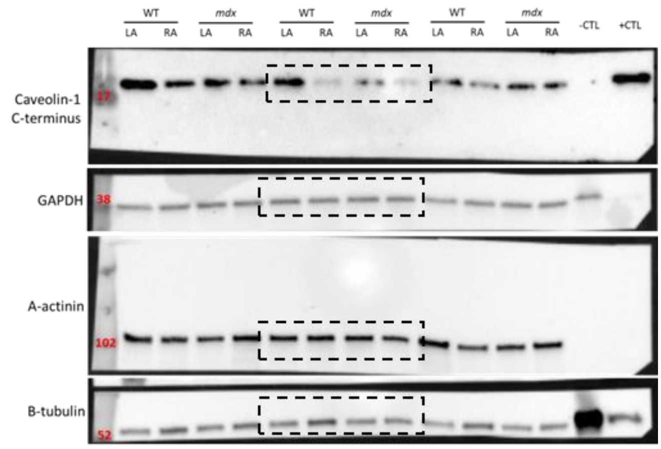

Supplementary Figure 19.

Uncropped membranes for Western blots presented in Supplementary Figure 10.
